# Supplementary material for: NLIMED: Natural Language Interface for Model Entity Discovery in Biosimulation Model Repositories
Source: Front Physiol. 2022 Feb 24;13:820683. doi: 10.3389/fphys.2022.820683 (PMC8908213; doi:10.3389/fphys.2022.820683)
Supplement: Supplementary file 1 [file Data_Sheet_1.PDF]

Supplementary Material

Table S1: Test Data: annotation of NLQ to ontology classes in the PMR

#	Query	Phrases	Annotation
1	sodium	sodium	http://purl.obolibrary.org/obo/CHEBI_29101
2	chloride	chloride	http://purl.obolibrary.org/obo/CHEBI_17996
3	hydron	hydron	http://purl.obolibrary.org/obo/CHEBI_15378
4	basolateral membrane	basolateral membrane	http://purl.obolibrary.org/obo/FMA_84669
5	apical plasma membrane	apical plasma membrane	http://purl.obolibrary.org/obo/FMA_84666
6	Portion of renal filtrate	portion of renal filtrate	http://purl.obolibrary.org/obo/FMA_280587
7	Collecting duct of renal tubule	collecting duct of renal tubule	http://purl.obolibrary.org/obo/FMA_15628
8	Epithelial cell of proximal tubule	epithelial cell of proximal tubule	http://purl.obolibrary.org/obo/FMA_70973
9	sodium/glucose cotransporter 1 (rat)	sodium/glucose cotransporter 1 (rat)	http://purl.obolibrary.org/obo/PR_P53790
10	give me model containing glucose transporter	glucose transporter	http://identifiers.org/go/GO:0005355
11	chemical concentration flow rate	chemical concentration flow rate	http://identifiers.org/opb/OPB_00593
12	voltage gated calcium channel complex	voltage gated calcium channel complex	http://identifiers.org/go/GO:0005891
13	van't Hoff law	van't hoff law	http://identifiers.org/opb/OPB_01061
14	left ventricular wall	left ventricular wall	http://purl.obolibrary.org/obo/FMA_9556
15	oxidative phosphorylation	oxidative phosphorylation	http://identifiers.org/go/GO:0006119
16	Cardiac myocyte	cardiac myocyte	http://purl.obolibrary.org/obo/FMA_14067
17	calcium driven ADP phosphorylation	calcium	http://identifiers.org/chebi/CHEBI:29108
		ADP phosphorylation	http://identifiers.org/go/GO:0006757
18	electrical potential difference across blood cell	electrical potential difference	http://identifiers.org/opb/OPB_00506
		blood cell	http://purl.obolibrary.org/obo/FMA_9670
19	mechanisms of SGLT2 inhibitors in lowering cytosolic calcium ion	sglt2 inhibitors	http://identifiers.org/chebi/CHEBI:73273
		cytosolic calcium ion transport	http://identifiers.org/go/GO:0060401
20	transport flux of potassium	flux	http://identifiers.org/opb/OPB_00593
		potassium	http://purl.obolibrary.org/obo/CHEBI_29103
21	concentration of sodium	concentration	http://identifiers.org/opb/OPB_00340
		sodium	http://purl.obolibrary.org/obo/CHEBI_29101
22	Nernst reversal potential of sodium	nernst reversal potential	http://identifiers.org/opb/OPB_01581
		sodium	http://purl.obolibrary.org/obo/CHEBI_29101
23	sodium flux	sodium	http://purl.obolibrary.org/obo/CHEBI_29101
		flux	http://identifiers.org/opb/OPB_00593
24	potassium concentration	potassium	http://purl.obolibrary.org/obo/CHEBI_29103
		concentration	http://identifiers.org/opb/OPB_00340
25	luminal antiporter activity	luminal	http://purl.obolibrary.org/obo/FMA_74550
		antiporter activity	http://identifiers.org/go/GO:0015297
26	electrical flow rate incorporating calcium efflux ATPase	electrical flow rate	http://identifiers.org/opb/OPB_00318

		calcium efflux ATPase	http://identifiers.org/go/GO:0005388
27	the mechanisms of calcium channel blocker in ventricular cardiac muscle cell	calcium channel blocker	http://identifiers.org/chebi/CHEBI:38215
		ventricular cardiac muscle cell	https://identifiers.org/cl/CL:2000046
28	sodium channel in renal intercalated cell	sodium channel	http://purl.obolibrary.org/obo/GO_0005272
		renal intercalated cell	http://identifiers.org/cl/CL:0005010
29	calcium ion significance in mitochondria	calcium ion	http://purl.obolibrary.org/obo/CHEBI_39124
		mitochondria	http://identifiers.org/go/GO:0005739
30	the regulation of mitochondria Na+/Ca2+ antiporter across plasma membrane	the regulation of mitochondria Na+/Ca2+ antiporter	http://identifiers.org/go/GO:0005432
		plasma membrane	http://purl.obolibrary.org/obo/FMA_63841
31	membrane hyperpolarization removes calcium channel blocker	membrane hyperpolarization	http://identifiers.org/go/GO:0060081
		calcium channel blocker	http://identifiers.org/chebi/CHEBI:38215
32	portion of cytosol in epithelial cell of distal tubule via sodium/hydrogen exchanger 3 (human)	portion of cytosol	http://purl.obolibrary.org/obo/FMA_66836
		epithelial cell of distal tubule	http://purl.obolibrary.org/obo/FMA_70981
		sodium/hydrogen exchanger 3 (human)	http://purl.obolibrary.org/obo/PR_P48764
33	glucose transporter from a kidney that counts for diabetes	glucose transporter	http://identifiers.org/go/GO:0005355
		kidney	http://purl.obolibrary.org/obo/FMA_7203
		diabetes	http://identifiers.org/chebi/CHEBI:5931
34	Flux of sodium through basolateral plasma membrane	flux	http://identifiers.org/opb/OPB_00593
		sodium	http://purl.obolibrary.org/obo/CHEBI_29101
		basolateral plasma membrane	http://purl.obolibrary.org/obo/FMA_84669
35	The sodium flow across the apical plasma membrane	sodium	http://purl.obolibrary.org/obo/CHEBI_29101
		flow	http://identifiers.org/opb/OPB_00593
		apical plasma membrane	http://purl.obolibrary.org/obo/FMA_84666
36	The exchange of chloride with bicarbonate across plasma membrane	chloride	http://purl.obolibrary.org/obo/CHEBI_17996
		bicarbonate	http://purl.obolibrary.org/obo/CHEBI_17544
		plasma membrane	http://purl.obolibrary.org/obo/FMA_63841
37	concentration of hydron in epithelial cell of proximal tubule compartment	concentration	http://identifiers.org/opb/OPB_00340
		hydron	http://purl.obolibrary.org/obo/CHEBI_15378
		epithelial cell of proximal tubule compartment	http://purl.obolibrary.org/obo/FMA_70973
38	NBCe1 transports H+ and HCO3-	NBCe1	http://purl.obolibrary.org/obo/PR_000015160
		H+	http://purl.obolibrary.org/obo/CHEBI_15378
		HCO3-	http://purl.obolibrary.org/obo/CHEBI_17544
39	the reverse mode of SGLT1 applies with of a-methyl-alpha-D-glucopyranoside in apical plasma membrane	SGLT1	http://purl.obolibrary.org/obo/PR_000015165
		a-methyl-alpha-D-glucopyranoside	http://identifiers.org/chebi/CHEBI:320061
		tissue fluid	http://purl.obolibrary.org/obo/FMA_84666
40	Na/H exchanger 3 rat and anion exchanger 1 rat in the distal convoluted tubule	Na/H exchanger 3	http://purl.obolibrary.org/obo/PR_P26433
		anion exchanger	http://purl.obolibrary.org/obo/PR_P23562
		distal convoluted tubule	http://identifiers.org/fma/FMA:17721
41	calcium channel complex and ryanodine receptor in cardiac myocite	calcium channel complex	http://identifiers.org/go/GO:0034704
		ryanodine receptor	http://identifiers.org/fma/FMA:62492
		cardiac myocite	http://purl.obolibrary.org/obo/FMA_14067
42	Flow rate of Calcium across sarcoplasmic reticulum membrane	flow rate	http://identifiers.org/opb/OPB_00593
		calcium	http://purl.obolibrary.org/obo/CHEBI_39124

		sarcoplasmic reticulum membrane	http://identifiers.org/go/GO:0033017
43	RyR2 (human) and IP3 related to sarcoplasmic reticulum calcium (human)	RyR2 (human)	http://purl.obolibrary.org/obo/PR_Q92736
		IP3	http://purl.obolibrary.org/obo/CHEBI_16595
		sarcoplasmic reticulum calcium (human)	http://purl.obolibrary.org/obo/PR_P16615
44	All entities containing Mitochondrial Tricarboxylic Acid Cycle, Oxidative Phosphorylation, and isocitrate dehydrogenase complex	mitochondrial tricarboxylic acid cycle	http://identifiers.org/go/GO:0030062
		oxidative phosphorylation	http://identifiers.org/go/GO:0006119
		isocitrate dehydrogenase complex	http://identifiers.org/go/GO:0005962
45	The calcium state incorporating L-type calcium channel and ryanodine receptor (human)	calcium	http://identifiers.org/chebi/CHEBI:22984
		L-type calcium channel	http://identifiers.org/go/GO:0008331
		ryanodine receptor (human)	http://purl.obolibrary.org/obo/PR_Q92736
46	Concentration of H+ in the tissue fluid	concentration	http://identifiers.org/opb/OPB_00340
		H+	http://purl.obolibrary.org/obo/CHEBI_15378
		tissue fluid	http://purl.obolibrary.org/obo/FMA_9673
47	flux of sodium across apical plasma membrane of the distal convoluted tubule	flux	http://identifiers.org/opb/OPB_00593
		sodium	http://purl.obolibrary.org/obo/CHEBI_29101
		apical plasma membrane	http://purl.obolibrary.org/obo/FMA_84666
		distal convoluted tubule	http://purl.obolibrary.org/obo/FMA_17721
48	flux of sodium across apical plasma membrane of the proximal convoluted tubule	flux	http://identifiers.org/opb/OPB_00593
		sodium	http://purl.obolibrary.org/obo/CHEBI_29101
		apical plasma membrane	http://purl.obolibrary.org/obo/FMA_84666
		proximal convoluted tubule	http://purl.obolibrary.org/obo/FMA_17693
49	flux of IP3 receptor through P2Y2 purinoceptor and apical plasma membrane	flux	http://identifiers.org/opb/OPB_00593
		ip3 receptor	http://purl.obolibrary.org/obo/CHEBI_131186
		p2y2 purinoceptor	http://purl.obolibrary.org/obo/PR_P35383
		apical plasma membrane	http://purl.obolibrary.org/obo/FMA_84666
50	concentration of sodium in the portion of cytosol in epithelial cell of distal tubule	concentration	http://identifiers.org/opb/OPB_00340
		sodium	http://purl.obolibrary.org/obo/CHEBI_29101
		portion of cytosol	http://purl.obolibrary.org/obo/FMA_66836
		epithelial cell of distal tubule	http://purl.obolibrary.org/obo/FMA_70981
51	sodium concentration in tissue fluid in epithelial cell of distal tubule	sodium	http://purl.obolibrary.org/obo/CHEBI_29101
		concentration	http://identifiers.org/opb/OPB_00340
		tissue fluid	http://purl.obolibrary.org/obo/FMA_9673
		epithelial cell of distal tubule	http://purl.obolibrary.org/obo/FMA_70981
52	K+ flow through potassium channel complex across apical cell membrane	K+	http://identifiers.org/chebi/CHEBI:29103
		flow	http://identifiers.org/opb/OPB_00593
		potassium channel complex	http://identifiers.org/go/GO:0034705
		apical cell membrane	http://purl.obolibrary.org/obo/FMA_84666

Table S2: Historical query and result models in the PMR

#	Query	Model
1	electrical potential difference human	hodgkin_huxley_1952/rawfile/HEAD/hodgkin_huxley_1952.cellml
		hodgkin_huxley_1952/rawfile/HEAD/hodgkin_huxley_1952_variant01.cellml
		baylor_hollingworth_chandler_2002/rawfile/HEAD/baylor_hollingworth_chandler_2002_b.cellml

2	electrical potential	

		baylor_hollingworth_chandler_2002/rawfile/HEAD/baylor_hollingworth_chandler_2002_d.cellml
		luo_rudy_1994/rawfile/HEAD/luo_rudy_1994.cellml
		colegrove_albrecht_friel_2000/rawfile/HEAD/colegrove_albrecht_friel_2000.cellml
		dougherty_wright_yew_2005/rawfile/HEAD/dougherty_wright_yew_2005.cellml
		yamaguchi_takaki_matsubara_yasuhara_suga_1996/rawfile/HEAD/yamaguchi_takaki_matsubara_yasuhara_suga_1996.cellml
		boyett_zhang_garny_holden_2001/rawfile/HEAD/boyett_zhang_garny_holden_2001.cellml
		iribe_kohl_noble_2006/rawfile/HEAD/iribe_kohl_noble_2006.cellml
		izakov_katsnelson_blyakhman_markhasin_shkylar_1991/rawfile/HEAD/izakov_katsnelson_blyakhman_markhasin_shkylar_1991.cellml
		stern_song_sham_yang_boheler_rios_1999/rawfile/HEAD/stern_song_sham_yang_boheler_rios_1999.cellml
		devries_sherman_2000/rawfile/HEAD/devries_sherman_2000.cellml
		marhl_haberichter_brumen_heinrich_2000/rawfile/HEAD/marhl_haberichter_brumen_heinrich_2000.cellml
3	heart tissue	tentusscher_noble_noble_panfilov_2004/rawfile/HEAD/tentusscher_noble_noble_panfilov_2004_c.cellml
		tentusscher_noble_noble_panfilov_2004/rawfile/HEAD/tentusscher_noble_noble_panfilov_2004_b.cellml
		tentusscher_noble_noble_panfilov_2004/rawfile/HEAD/tentusscher_noble_noble_panfilov_2004_a.cellml
4	calmodulin	baylor_hollingworth_chandler_2002/rawfile/HEAD/baylor_hollingworth_chandler_2002_b.cellml
		baylor_hollingworth_chandler_2002/rawfile/HEAD/baylor_hollingworth_chandler_2002_d.cellml
		luo_rudy_1994/rawfile/HEAD/luo_rudy_1994.cellml
		colegrove_albrecht_friel_2000/rawfile/HEAD/colegrove_albrecht_friel_2000.cellml
		dougherty_wright_yew_2005/rawfile/HEAD/dougherty_wright_yew_2005.cellml
		yamaguchi_takaki_matsubara_yasuhara_suga_1996/rawfile/HEAD/yamaguchi_takaki_matsubara_yasuhara_suga_1996.cellml
		boyett_zhang_garny_holden_2001/rawfile/HEAD/boyett_zhang_garny_holden_2001.cellml
		iribe_kohl_noble_2006/rawfile/HEAD/iribe_kohl_noble_2006.cellml
		izakov_katsnelson_blyakhman_markhasin_shkylar_1991/rawfile/HEAD/izakov_katsnelson_blyakhman_markhasin_shkylar_1991.cellml
		stern_song_sham_yang_boheler_rios_1999/rawfile/HEAD/stern_song_sham_yang_boheler_rios_1999.cellml
		devries_sherman_2000/rawfile/HEAD/devries_sherman_2000.cellml
		marhl_haberichter_brumen_heinrich_2000/rawfile/HEAD/marhl_haberichter_brumen_heinrich_2000.cellml
5	a synthetic oscillatory network of transcriptional	baylor_hollingworth_chandler_2002/rawfile/HEAD/baylor_hollingworth_chandler_2002_b.cellml
		baylor_hollingworth_chandler_2002/rawfile/HEAD/baylor_hollingworth_chandler_2002_d.cellml
		luo_rudy_1994/rawfile/HEAD/luo_rudy_1994.cellml
		colegrove_albrecht_friel_2000/rawfile/HEAD/colegrove_albrecht_friel_2000.cellml
		dougherty_wright_yew_2005/rawfile/HEAD/dougherty_wright_yew_2005.cellml
		yamaguchi_takaki_matsubara_yasuhara_suga_1996/rawfile/HEAD/yamaguchi_takaki_matsubara_yasuhara_suga_1996.cellml
		boyett_zhang_garny_holden_2001/rawfile/HEAD/boyett_zhang_garny_holden_2001.cellml
		iribe_kohl_noble_2006/rawfile/HEAD/iribe_kohl_noble_2006.cellml
		izakov_katsnelson_blyakhman_markhasin_shkylar_1991/rawfile/HEAD/izakov_katsnelson_blyakhman_markhasin_shkylar_1991.cellml
		stern_song_sham_yang_boheler_rios_1999/rawfile/HEAD/stern_song_sham_yang_boheler_rios_1999.cellml
		devries_sherman_2000/rawfile/HEAD/devries_sherman_2000.cellml
		marhl_haberichter_brumen_heinrich_2000/rawfile/HEAD/marhl_haberichter_brumen_heinrich_2000.cellml
6	heart	baylor_hollingworth_chandler_2002/rawfile/HEAD/baylor_hollingworth_chandler_2002_b.cellml
		baylor_hollingworth_chandler_2002/rawfile/HEAD/baylor_hollingworth_chandler_2002_d.cellml
		luo_rudy_1994/rawfile/HEAD/luo_rudy_1994.cellml
		colegrove_albrecht_friel_2000/rawfile/HEAD/colegrove_albrecht_friel_2000.cellml
		dougherty_wright_yew_2005/rawfile/HEAD/dougherty_wright_yew_2005.cellml
		yamaguchi_takaki_matsubara_yasuhara_suga_1996/rawfile/HEAD/yamaguchi_takaki_matsubara_yasuhara_suga_1996.cellml
		boyett_zhang_garny_holden_2001/rawfile/HEAD/boyett_zhang_garny_holden_2001.cellml
		iribe_kohl_noble_2006/rawfile/HEAD/iribe_kohl_noble_2006.cellml
		izakov_katsnelson_blyakhman_markhasin_shkylar_1991/rawfile/HEAD/izakov_katsnelson_blyakhman_markhasin_shkylar_1991.cellml
		stern_song_sham_yang_boheler_rios_1999/rawfile/HEAD/stern_song_sham_yang_boheler_rios_1999.cellml
		devries_sherman_2000/rawfile/HEAD/devries_sherman_2000.cellml
		marhl_haberichter_brumen_heinrich_2000/rawfile/HEAD/marhl_haberichter_brumen_heinrich_2000.cellml
7	ion	michailova_mcculloch_2001/rawfile/HEAD/michailova_mcculloch_2001.cellml
		jafri_rice_winslow_1998/rawfile/HEAD/jafri_rice_winslow_1998_a.cellml
		winslow_rice_jafri_marban_ororke_1999/rawfile/HEAD/winslow_rice_jafri_marban_ororke_1999.cellml
		jafri_rice_winslow_1998/rawfile/HEAD/jafri_rice_winslow_1998_b.cellml
8	minimal haemodynamic system model including ventricular interaction and valve dynamics	baylor_hollingworth_chandler_2002/rawfile/HEAD/baylor_hollingworth_chandler_2002_b.cellml
		baylor_hollingworth_chandler_2002/rawfile/HEAD/baylor_hollingworth_chandler_2002_d.cellml
		luo_rudy_1994/rawfile/HEAD/luo_rudy_1994.cellml
		colegrove_albrecht_friel_2000/rawfile/HEAD/colegrove_albrecht_friel_2000.cellml
		dougherty_wright_yew_2005/rawfile/HEAD/dougherty_wright_yew_2005.cellml
		yamaguchi_takaki_matsubara_yasuhara_suga_1996/rawfile/HEAD/yamaguchi_takaki_matsubara_yasuhara_suga_1996.cellml

		boyett_zhang_garny_holden_2001/rawfile/HEAD/boyett_zhang_garny_holden_2001.cellml
		iribe_kohl_noble_2006/rawfile/HEAD/iribe_kohl_noble_2006.cellml
		izakov_katsnelson_blyakhman_markhasin_shkylar_1991/rawfile/HEAD/izakov_katsnelson_blyakhman_markhasin_shkylar_1991.cellml
		stern_song_sham_yang_boheler_rios_1999/rawfile/HEAD/stern_song_sham_yang_boheler_rios_1999.cellml
		devries_sherman_2000/rawfile/HEAD/devries_sherman_2000.cellml
		marhl_haberichter_brumen_heinrich_2000/rawfile/HEAD/marhl_haberichter_brumen_heinrich_2000.cellml
9	diffusion	keener_2001/rawfile/HEAD/keener_2001.cellml
10	calmodulin mediates differential sensitivity of camkii and calcineurin to local	baylor_hollingworth_chandler_2002/rawfile/HEAD/baylor_hollingworth_chandler_2002_b.cellml
		baylor_hollingworth_chandler_2002/rawfile/HEAD/baylor_hollingworth_chandler_2002_d.cellml
		luo_rudy_1994/rawfile/HEAD/luo_rudy_1994.cellml
		colegrove_albrecht_friel_2000/rawfile/HEAD/colegrove_albrecht_friel_2000.cellml
		dougherty_wright_yew_2005/rawfile/HEAD/dougherty_wright_yew_2005.cellml
		yamaguchi_takaki_matsubara_yasuhara_suga_1996/rawfile/HEAD/yamaguchi_takaki_matsubara_yasuhara_suga_1996.cellml
		boyett_zhang_garny_holden_2001/rawfile/HEAD/boyett_zhang_garny_holden_2001.cellml
		iribe_kohl_noble_2006/rawfile/HEAD/iribe_kohl_noble_2006.cellml
		izakov_katsnelson_blyakhman_markhasin_shkylar_1991/rawfile/HEAD/izakov_katsnelson_blyakhman_markhasin_shkylar_1991.cellml
		stern_song_sham_yang_boheler_rios_1999/rawfile/HEAD/stern_song_sham_yang_boheler_rios_1999.cellml
		devries_sherman_2000/rawfile/HEAD/devries_sherman_2000.cellml
		marhl_haberichter_brumen_heinrich_2000/rawfile/HEAD/marhl_haberichter_brumen_heinrich_2000.cellml
11	a mathematical treatment of integrated ca dynamics within the ventricular myocyte	shannon_wang_puglisi_weber_bers_2004/rawfile/HEAD/shannon_wang_puglisi_weber_bers_2004_a.cellml
		shannon_wang_puglisi_weber_bers_2004/rawfile/HEAD/shannon_wang_puglisi_weber_bers_2004_b.cellml
12	human atrial	baylor_hollingworth_chandler_2002/rawfile/HEAD/baylor_hollingworth_chandler_2002_b.cellml
		baylor_hollingworth_chandler_2002/rawfile/HEAD/baylor_hollingworth_chandler_2002_d.cellml
		luo_rudy_1994/rawfile/HEAD/luo_rudy_1994.cellml
		colegrove_albrecht_friel_2000/rawfile/HEAD/colegrove_albrecht_friel_2000.cellml
		dougherty_wright_yew_2005/rawfile/HEAD/dougherty_wright_yew_2005.cellml
		yamaguchi_takaki_matsubara_yasuhara_suga_1996/rawfile/HEAD/yamaguchi_takaki_matsubara_yasuhara_suga_1996.cellml
		boyett_zhang_garny_holden_2001/rawfile/HEAD/boyett_zhang_garny_holden_2001.cellml
		iribe_kohl_noble_2006/rawfile/HEAD/iribe_kohl_noble_2006.cellml
		izakov_katsnelson_blyakhman_markhasin_shkylar_1991/rawfile/HEAD/izakov_katsnelson_blyakhman_markhasin_shkylar_1991.cellml
		stern_song_sham_yang_boheler_rios_1999/rawfile/HEAD/stern_song_sham_yang_boheler_rios_1999.cellml
		devries_sherman_2000/rawfile/HEAD/devries_sherman_2000.cellml
		marhl_haberichter_brumen_heinrich_2000/rawfile/HEAD/marhl_haberichter_brumen_heinrich_2000.cellml
13	thomas r. shannon	shannon_wang_puglisi_weber_bers_2004/rawfile/HEAD/shannon_wang_puglisi_weber_bers_2004_b.cellml
		shannon_wang_puglisi_weber_bers_2004/rawfile/HEAD/shannon_wang_puglisi_weber_bers_2004_a.cellml
14	atrial fibrillation	baylor_hollingworth_chandler_2002/rawfile/HEAD/baylor_hollingworth_chandler_2002_b.cellml
		baylor_hollingworth_chandler_2002/rawfile/HEAD/baylor_hollingworth_chandler_2002_d.cellml
		luo_rudy_1994/rawfile/HEAD/luo_rudy_1994.cellml
		colegrove_albrecht_friel_2000/rawfile/HEAD/colegrove_albrecht_friel_2000.cellml
		dougherty_wright_yew_2005/rawfile/HEAD/dougherty_wright_yew_2005.cellml
		yamaguchi_takaki_matsubara_yasuhara_suga_1996/rawfile/HEAD/yamaguchi_takaki_matsubara_yasuhara_suga_1996.cellml
		boyett_zhang_garny_holden_2001/rawfile/HEAD/boyett_zhang_garny_holden_2001.cellml
		iribe_kohl_noble_2006/rawfile/HEAD/iribe_kohl_noble_2006.cellml
		izakov_katsnelson_blyakhman_markhasin_shkylar_1991/rawfile/HEAD/izakov_katsnelson_blyakhman_markhasin_shkylar_1991.cellml
		stern_song_sham_yang_boheler_rios_1999/rawfile/HEAD/stern_song_sham_yang_boheler_rios_1999.cellml
		devries_sherman_2000/rawfile/HEAD/devries_sherman_2000.cellml
		marhl_haberichter_brumen_heinrich_2000/rawfile/HEAD/marhl_haberichter_brumen_heinrich_2000.cellml
15	nhe3	267/rawfile/HEAD/weinstein_1995-rabbit.cellml
		267/rawfile/HEAD/SEDML/weinstein_1995/weinstein_1995.cellml
		267/rawfile/HEAD/weinstein_1995-mouse.cellml
		267/rawfile/HEAD/weinstein_1995.cellml
16	caffeine concentration model	baylor_hollingworth_chandler_2002/rawfile/HEAD/baylor_hollingworth_chandler_2002_b.cellml
		baylor_hollingworth_chandler_2002/rawfile/HEAD/baylor_hollingworth_chandler_2002_d.cellml
		luo_rudy_1994/rawfile/HEAD/luo_rudy_1994.cellml
		colegrove_albrecht_friel_2000/rawfile/HEAD/colegrove_albrecht_friel_2000.cellml
		dougherty_wright_yew_2005/rawfile/HEAD/dougherty_wright_yew_2005.cellml
		yamaguchi_takaki_matsubara_yasuhara_suga_1996/rawfile/HEAD/yamaguchi_takaki_matsubara_yasuhara_suga_1996.cellml

		boyett_zhang_garny_holden_2001/rawfile/HEAD/boyett_zhang_garny_holden_2001.cellml
		iribe_kohl_noble_2006/rawfile/HEAD/iribe_kohl_noble_2006.cellml
		izakov_katsnelson_blyakhman_markhasin_shkylar_1991/rawfile/HEAD/izakov_katsnelson_blyakhman_markhasin_shkylar_1991.cellml
		stern_song_sham_yang_boheler_rios_1999/rawfile/HEAD/stern_song_sham_yang_boheler_rios_1999.cellml
		devries_sherman_2000/rawfile/HEAD/devries_sherman_2000.cellml
		marhl_haberichter_brumen_heinrich_2000/rawfile/HEAD/marhl_haberichter_brumen_heinrich_2000.cellml
17	gap junctions	bindschadler_sneyd_2001/rawfile/HEAD/bindschadler_sneyd_2001.cellml
		267/rawfile/HEAD/bindschadler_sneyd_2001.cellml
18	gap junction	baylor_hollingworth_chandler_2002/rawfile/HEAD/baylor_hollingworth_chandler_2002_b.cellml
		baylor_hollingworth_chandler_2002/rawfile/HEAD/baylor_hollingworth_chandler_2002_d.cellml
		luo_rudy_1994/rawfile/HEAD/luo_rudy_1994.cellml
		colegrove_albrecht_friel_2000/rawfile/HEAD/colegrove_albrecht_friel_2000.cellml
		dougherty_wright_yew_2005/rawfile/HEAD/dougherty_wright_yew_2005.cellml
		yamaguchi_takaki_matsubara_yasuhara_suga_1996/rawfile/HEAD/yamaguchi_takaki_matsubara_yasuhara_suga_1996.cellml
		boyett_zhang_garny_holden_2001/rawfile/HEAD/boyett_zhang_garny_holden_2001.cellml
		iribe_kohl_noble_2006/rawfile/HEAD/iribe_kohl_noble_2006.cellml
		izakov_katsnelson_blyakhman_markhasin_shkylar_1991/rawfile/HEAD/izakov_katsnelson_blyakhman_markhasin_shkylar_1991.cellml
		stern_song_sham_yang_boheler_rios_1999/rawfile/HEAD/stern_song_sham_yang_boheler_rios_1999.cellml
		devries_sherman_2000/rawfile/HEAD/devries_sherman_2000.cellml
		marhl_haberichter_brumen_heinrich_2000/rawfile/HEAD/marhl_haberichter_brumen_heinrich_2000.cellml
19	a novel computational model of the human ventricular action potential and ca transient	baylor_hollingworth_chandler_2002/rawfile/HEAD/baylor_hollingworth_chandler_2002_b.cellml
		baylor_hollingworth_chandler_2002/rawfile/HEAD/baylor_hollingworth_chandler_2002_d.cellml
		luo_rudy_1994/rawfile/HEAD/luo_rudy_1994.cellml
		colegrove_albrecht_friel_2000/rawfile/HEAD/colegrove_albrecht_friel_2000.cellml
		dougherty_wright_yew_2005/rawfile/HEAD/dougherty_wright_yew_2005.cellml
		yamaguchi_takaki_matsubara_yasuhara_suga_1996/rawfile/HEAD/yamaguchi_takaki_matsubara_yasuhara_suga_1996.cellml
		boyett_zhang_garny_holden_2001/rawfile/HEAD/boyett_zhang_garny_holden_2001.cellml
		iribe_kohl_noble_2006/rawfile/HEAD/iribe_kohl_noble_2006.cellml
		izakov_katsnelson_blyakhman_markhasin_shkylar_1991/rawfile/HEAD/izakov_katsnelson_blyakhman_markhasin_shkylar_1991.cellml
		stern_song_sham_yang_boheler_rios_1999/rawfile/HEAD/stern_song_sham_yang_boheler_rios_1999.cellml
		devries_sherman_2000/rawfile/HEAD/devries_sherman_2000.cellml
		marhl_haberichter_brumen_heinrich_2000/rawfile/HEAD/marhl_haberichter_brumen_heinrich_2000.cellml
20	a model for human ventricular tissue	tentusscher_noble_noble_panfilov_2004/rawfile/HEAD/tentusscher_noble_noble_panfilov_2004_c.cellml
		tentusscher_noble_noble_panfilov_2004/rawfile/HEAD/tentusscher_noble_noble_panfilov_2004_b.cellml
		tentusscher_noble_noble_panfilov_2004/rawfile/HEAD/tentusscher_noble_noble_panfilov_2004_a.cellml
21	a model of the ventricular cardiac action potential: depolarization, repolarization, and their interaction	luo_rudy_1991/rawfile/HEAD/luo_rudy_1991.cellml
22	cardiomyocyte	baylor_hollingworth_chandler_2002/rawfile/HEAD/baylor_hollingworth_chandler_2002_b.cellml
		baylor_hollingworth_chandler_2002/rawfile/HEAD/baylor_hollingworth_chandler_2002_d.cellml
		luo_rudy_1994/rawfile/HEAD/luo_rudy_1994.cellml
		colegrove_albrecht_friel_2000/rawfile/HEAD/colegrove_albrecht_friel_2000.cellml
		dougherty_wright_yew_2005/rawfile/HEAD/dougherty_wright_yew_2005.cellml
		yamaguchi_takaki_matsubara_yasuhara_suga_1996/rawfile/HEAD/yamaguchi_takaki_matsubara_yasuhara_suga_1996.cellml
		boyett_zhang_garny_holden_2001/rawfile/HEAD/boyett_zhang_garny_holden_2001.cellml
		iribe_kohl_noble_2006/rawfile/HEAD/iribe_kohl_noble_2006.cellml
		izakov_katsnelson_blyakhman_markhasin_shkylar_1991/rawfile/HEAD/izakov_katsnelson_blyakhman_markhasin_shkylar_1991.cellml
		stern_song_sham_yang_boheler_rios_1999/rawfile/HEAD/stern_song_sham_yang_boheler_rios_1999.cellml
		devries_sherman_2000/rawfile/HEAD/devries_sherman_2000.cellml
		marhl_haberichter_brumen_heinrich_2000/rawfile/HEAD/marhl_haberichter_brumen_heinrich_2000.cellml
23	a mathematical model of plasma membrane electrophysiology and calcium dynamics in vascular endothelial cells python model	baylor_hollingworth_chandler_2002/rawfile/HEAD/baylor_hollingworth_chandler_2002_b.cellml
		baylor_hollingworth_chandler_2002/rawfile/HEAD/baylor_hollingworth_chandler_2002_d.cellml
		luo_rudy_1994/rawfile/HEAD/luo_rudy_1994.cellml
		colegrove_albrecht_friel_2000/rawfile/HEAD/colegrove_albrecht_friel_2000.cellml
		dougherty_wright_yew_2005/rawfile/HEAD/dougherty_wright_yew_2005.cellml
		yamaguchi_takaki_matsubara_yasuhara_suga_1996/rawfile/HEAD/yamaguchi_takaki_matsubara_yasuhara_suga_1996.cellml
		boyett_zhang_garny_holden_2001/rawfile/HEAD/boyett_zhang_garny_holden_2001.cellml
		iribe_kohl_noble_2006/rawfile/HEAD/iribe_kohl_noble_2006.cellml
		izakov_katsnelson_blyakhman_markhasin_shkylar_1991/rawfile/HEAD/izakov_katsnelson_blyakhman_markhasin_shkylar_1991.cellml
		stern_song_sham_yang_boheler_rios_1999/rawfile/HEAD/stern_song_sham_yang_boheler_rios_1999.cellml

		devries_sherman_2000/rawfile/HEAD/devries_sherman_2000.cellml
		marhl_haberichter_brumen_heinrich_2000/rawfile/HEAD/marhl_haberichter_brumen_heinrich_2000.cellml
24	cell cycle	baylor_hollingworth_chandler_2002/rawfile/HEAD/baylor_hollingworth_chandler_2002_b.cellml
		baylor_hollingworth_chandler_2002/rawfile/HEAD/baylor_hollingworth_chandler_2002_d.cellml
		luo_rudy_1994/rawfile/HEAD/luo_rudy_1994.cellml
		colegrove_albrecht_friel_2000/rawfile/HEAD/colegrove_albrecht_friel_2000.cellml
		dougherty_wright_yew_2005/rawfile/HEAD/dougherty_wright_yew_2005.cellml
		yamaguchi_takaki_matsubara_yasuhara_suga_1996/rawfile/HEAD/yamaguchi_takaki_matsubara_yasuhara_suga_1996.cellml
		boyett_zhang_garny_holden_2001/rawfile/HEAD/boyett_zhang_garny_holden_2001.cellml
		iribe_kohl_noble_2006/rawfile/HEAD/iribe_kohl_noble_2006.cellml
		izakov_katsnelson_blyakhman_markhasin_shkylar_1991/rawfile/HEAD/izakov_katsnelson_blyakhman_markhasin_shkylar_1991.cellml
		stern_song_sham_yang_boheler_rios_1999/rawfile/HEAD/stern_song_sham_yang_boheler_rios_1999.cellml
		devries_sherman_2000/rawfile/HEAD/devries_sherman_2000.cellml
		marhl_haberichter_brumen_heinrich_2000/rawfile/HEAD/marhl_haberichter_brumen_heinrich_2000.cellml
25	using physiome standards to couple cellular functions for rat cardiac excitation-contraction	terkildsen_niederer_crampin_hunter_smith_2008/rawfile/HEAD/Pandit_Hinch_Niederer.cellml
26	rat gattoni	terkildsen_niederer_crampin_hunter_smith_2008/rawfile/HEAD/Pandit_Hinch_Niederer.cellml
27	action potential	baylor_hollingworth_chandler_2002/rawfile/HEAD/baylor_hollingworth_chandler_2002_b.cellml
		baylor_hollingworth_chandler_2002/rawfile/HEAD/baylor_hollingworth_chandler_2002_d.cellml
		luo_rudy_1994/rawfile/HEAD/luo_rudy_1994.cellml
		colegrove_albrecht_friel_2000/rawfile/HEAD/colegrove_albrecht_friel_2000.cellml
		dougherty_wright_yew_2005/rawfile/HEAD/dougherty_wright_yew_2005.cellml
		yamaguchi_takaki_matsubara_yasuhara_suga_1996/rawfile/HEAD/yamaguchi_takaki_matsubara_yasuhara_suga_1996.cellml
		luo_rudy_1991/rawfile/HEAD/luo_rudy_1991.cellml
		boyett_zhang_garny_holden_2001/rawfile/HEAD/boyett_zhang_garny_holden_2001.cellml
		iribe_kohl_noble_2006/rawfile/HEAD/iribe_kohl_noble_2006.cellml
		izakov_katsnelson_blyakhman_markhasin_shkylar_1991/rawfile/HEAD/izakov_katsnelson_blyakhman_markhasin_shkylar_1991.cellml
		stern_song_sham_yang_boheler_rios_1999/rawfile/HEAD/stern_song_sham_yang_boheler_rios_1999.cellml
		devries_sherman_2000/rawfile/HEAD/devries_sherman_2000.cellml
		marhl_haberichter_brumen_heinrich_2000/rawfile/HEAD/marhl_haberichter_brumen_heinrich_2000.cellml
28	human fibroblast	baylor_hollingworth_chandler_2002/rawfile/HEAD/baylor_hollingworth_chandler_2002_b.cellml
		baylor_hollingworth_chandler_2002/rawfile/HEAD/baylor_hollingworth_chandler_2002_d.cellml
		luo_rudy_1994/rawfile/HEAD/luo_rudy_1994.cellml
		colegrove_albrecht_friel_2000/rawfile/HEAD/colegrove_albrecht_friel_2000.cellml
		dougherty_wright_yew_2005/rawfile/HEAD/dougherty_wright_yew_2005.cellml
		yamaguchi_takaki_matsubara_yasuhara_suga_1996/rawfile/HEAD/yamaguchi_takaki_matsubara_yasuhara_suga_1996.cellml
		boyett_zhang_garny_holden_2001/rawfile/HEAD/boyett_zhang_garny_holden_2001.cellml
		iribe_kohl_noble_2006/rawfile/HEAD/iribe_kohl_noble_2006.cellml
		izakov_katsnelson_blyakhman_markhasin_shkylar_1991/rawfile/HEAD/izakov_katsnelson_blyakhman_markhasin_shkylar_1991.cellml
		stern_song_sham_yang_boheler_rios_1999/rawfile/HEAD/stern_song_sham_yang_boheler_rios_1999.cellml
		devries_sherman_2000/rawfile/HEAD/devries_sherman_2000.cellml
		marhl_haberichter_brumen_heinrich_2000/rawfile/HEAD/marhl_haberichter_brumen_heinrich_2000.cellml
29	minimal hemodynamics heart	baylor_hollingworth_chandler_2002/rawfile/HEAD/baylor_hollingworth_chandler_2002_b.cellml
		baylor_hollingworth_chandler_2002/rawfile/HEAD/baylor_hollingworth_chandler_2002_d.cellml
		luo_rudy_1994/rawfile/HEAD/luo_rudy_1994.cellml
		colegrove_albrecht_friel_2000/rawfile/HEAD/colegrove_albrecht_friel_2000.cellml
		dougherty_wright_yew_2005/rawfile/HEAD/dougherty_wright_yew_2005.cellml
		yamaguchi_takaki_matsubara_yasuhara_suga_1996/rawfile/HEAD/yamaguchi_takaki_matsubara_yasuhara_suga_1996.cellml
		boyett_zhang_garny_holden_2001/rawfile/HEAD/boyett_zhang_garny_holden_2001.cellml
		iribe_kohl_noble_2006/rawfile/HEAD/iribe_kohl_noble_2006.cellml
		izakov_katsnelson_blyakhman_markhasin_shkylar_1991/rawfile/HEAD/izakov_katsnelson_blyakhman_markhasin_shkylar_1991.cellml
		stern_song_sham_yang_boheler_rios_1999/rawfile/HEAD/stern_song_sham_yang_boheler_rios_1999.cellml
		devries_sherman_2000/rawfile/HEAD/devries_sherman_2000.cellml
		marhl_haberichter_brumen_heinrich_2000/rawfile/HEAD/marhl_haberichter_brumen_heinrich_2000.cellml
30	di francesco-noble	difrancesco_noble_1985/rawfile/HEAD/difrancesco_noble_1985.cellml
		baylor_hollingworth_chandler_2002/rawfile/HEAD/baylor_hollingworth_chandler_2002_b.cellml
		baylor_hollingworth_chandler_2002/rawfile/HEAD/baylor_hollingworth_chandler_2002_d.cellml
		luo_rudy_1994/rawfile/HEAD/luo_rudy_1994.cellml
		colegrove_albrecht_friel_2000/rawfile/HEAD/colegrove_albrecht_friel_2000.cellml
		dougherty_wright_yew_2005/rawfile/HEAD/dougherty_wright_yew_2005.cellml
31	smith, crampin	

		yamaguchi_takaki_matsubara_yasuhara_suga_1996/rawfile/HEAD/yamaguchi_takaki_matsubara_yasuhara_suga_1996.cellml
		boyett_zhang_garny_holden_2001/rawfile/HEAD/boyett_zhang_garny_holden_2001.cellml
		iribe_kohl_noble_2006/rawfile/HEAD/iribe_kohl_noble_2006.cellml
		izakov_katsnelson_blyakhman_markhasin_shkylar_1991/rawfile/HEAD/izakov_katsnelson_blyakhman_markhasin_shkylar_1991.cellml
		stern_song_sham_yang_boheler_rios_1999/rawfile/HEAD/stern_song_sham_yang_boheler_rios_1999.cellml
		devries_sherman_2000/rawfile/HEAD/devries_sherman_2000.cellml
		marhl_haberichter_brumen_heinrich_2000/rawfile/HEAD/marhl_haberichter_brumen_heinrich_2000.cellml
32	modeling the effects of caffeine on blood pressure	baylor_hollingworth_chandler_2002/rawfile/HEAD/baylor_hollingworth_chandler_2002_b.cellml
		baylor_hollingworth_chandler_2002/rawfile/HEAD/baylor_hollingworth_chandler_2002_d.cellml
		luo_rudy_1994/rawfile/HEAD/luo_rudy_1994.cellml
		colegrove_albrecht_friel_2000/rawfile/HEAD/colegrove_albrecht_friel_2000.cellml
		dougherty_wright_yew_2005/rawfile/HEAD/dougherty_wright_yew_2005.cellml
		yamaguchi_takaki_matsubara_yasuhara_suga_1996/rawfile/HEAD/yamaguchi_takaki_matsubara_yasuhara_suga_1996.cellml
		boyett_zhang_garny_holden_2001/rawfile/HEAD/boyett_zhang_garny_holden_2001.cellml
		iribe_kohl_noble_2006/rawfile/HEAD/iribe_kohl_noble_2006.cellml
		izakov_katsnelson_blyakhman_markhasin_shkylar_1991/rawfile/HEAD/izakov_katsnelson_blyakhman_markhasin_shkylar_1991.cellml
		stern_song_sham_yang_boheler_rios_1999/rawfile/HEAD/stern_song_sham_yang_boheler_rios_1999.cellml
		devries_sherman_2000/rawfile/HEAD/devries_sherman_2000.cellml
		marhl_haberichter_brumen_heinrich_2000/rawfile/HEAD/marhl_haberichter_brumen_heinrich_2000.cellml
33	cardiac*	baylor_hollingworth_chandler_2002/rawfile/HEAD/baylor_hollingworth_chandler_2002_b.cellml
		baylor_hollingworth_chandler_2002/rawfile/HEAD/baylor_hollingworth_chandler_2002_d.cellml
		luo_rudy_1994/rawfile/HEAD/luo_rudy_1994.cellml
		colegrove_albrecht_friel_2000/rawfile/HEAD/colegrove_albrecht_friel_2000.cellml
		dougherty_wright_yew_2005/rawfile/HEAD/dougherty_wright_yew_2005.cellml
		yamaguchi_takaki_matsubara_yasuhara_suga_1996/rawfile/HEAD/yamaguchi_takaki_matsubara_yasuhara_suga_1996.cellml
		boyett_zhang_garny_holden_2001/rawfile/HEAD/boyett_zhang_garny_holden_2001.cellml
		iribe_kohl_noble_2006/rawfile/HEAD/iribe_kohl_noble_2006.cellml
		izakov_katsnelson_blyakhman_markhasin_shkylar_1991/rawfile/HEAD/izakov_katsnelson_blyakhman_markhasin_shkylar_1991.cellml
		stern_song_sham_yang_boheler_rios_1999/rawfile/HEAD/stern_song_sham_yang_boheler_rios_1999.cellml
		devries_sherman_2000/rawfile/HEAD/devries_sherman_2000.cellml
		marhl_haberichter_brumen_heinrich_2000/rawfile/HEAD/marhl_haberichter_brumen_heinrich_2000.cellml
34	insulin	baylor_hollingworth_chandler_2002/rawfile/HEAD/baylor_hollingworth_chandler_2002_b.cellml
		baylor_hollingworth_chandler_2002/rawfile/HEAD/baylor_hollingworth_chandler_2002_d.cellml
		luo_rudy_1994/rawfile/HEAD/luo_rudy_1994.cellml
		gall_susa_1999/rawfile/HEAD/gall_susa_1999_b.cellml
		colegrove_albrecht_friel_2000/rawfile/HEAD/colegrove_albrecht_friel_2000.cellml
		dougherty_wright_yew_2005/rawfile/HEAD/dougherty_wright_yew_2005.cellml
		gall_susa_1999/rawfile/HEAD/gall_susa_1999_c.cellml
		yamaguchi_takaki_matsubara_yasuhara_suga_1996/rawfile/HEAD/yamaguchi_takaki_matsubara_yasuhara_suga_1996.cellml
		boyett_zhang_garny_holden_2001/rawfile/HEAD/boyett_zhang_garny_holden_2001.cellml
		chay_1997/rawfile/HEAD/chay_1997.cellml
		570/rawfile/HEAD/bertram_satin_pedersen_luciani_sherman_2007.cellml
		keener_2001/rawfile/HEAD/keener_2001.cellml
		iribe_kohl_noble_2006/rawfile/HEAD/iribe_kohl_noble_2006.cellml
		magnus_keizer_1998/rawfile/HEAD/magnus_keizer_1998.cellml
		goforth_bertram_khan_zhang_sherman_satin_2002/rawfile/HEAD/goforth_bertram_khan_zhang_sherman_satin_2002.cellml
		gall_susa_1999/rawfile/HEAD/gall_susa_1999_a.cellml
		izakov_katsnelson_blyakhman_markhasin_shkylar_1991/rawfile/HEAD/izakov_katsnelson_blyakhman_markhasin_shkylar_1991.cellml
		stern_song_sham_yang_boheler_rios_1999/rawfile/HEAD/stern_song_sham_yang_boheler_rios_1999.cellml
		devries_sherman_2000/rawfile/HEAD/devries_sherman_2000.cellml
		marhl_haberichter_brumen_heinrich_2000/rawfile/HEAD/marhl_haberichter_brumen_heinrich_2000.cellml
35	beta cell	bertram_previte_sherman_kinard_satin_2000/rawfile/HEAD/bertram_previte_sherman_kinard_satin_2000_medium.cellml
		bertram_previte_sherman_kinard_satin_2000/rawfile/HEAD/bertram_previte_sherman_kinard_satin_2000_fast.cellml
		bertram_previte_sherman_kinard_satin_2000/rawfile/HEAD/bertram_previte_sherman_kinard_satin_2000_slow.cellml
36	camkii	baylor_hollingworth_chandler_2002/rawfile/HEAD/baylor_hollingworth_chandler_2002_b.cellml
		baylor_hollingworth_chandler_2002/rawfile/HEAD/baylor_hollingworth_chandler_2002_d.cellml
		luo_rudy_1994/rawfile/HEAD/luo_rudy_1994.cellml
		colegrove_albrecht_friel_2000/rawfile/HEAD/colegrove_albrecht_friel_2000.cellml
		dougherty_wright_yew_2005/rawfile/HEAD/dougherty_wright_yew_2005.cellml
		yamaguchi_takaki_matsubara_yasuhara_suga_1996/rawfile/HEAD/yamaguchi_takaki_matsubara_yasuhara_suga_1996.cellml

		boyett_zhang_garny_holden_2001/rawfile/HEAD/boyett_zhang_garny_holden_2001.cellml
		iribe_kohl_noble_2006/rawfile/HEAD/iribe_kohl_noble_2006.cellml
		izakov_katsnelson_blyakhman_markhasin_shkylar_1991/rawfile/HEAD/izakov_katsnelson_blyakhman_markhasin_shkylar_1991.cellml
		stern_song_sham_yang_boheler_rios_1999/rawfile/HEAD/stern_song_sham_yang_boheler_rios_1999.cellml
		devries_sherman_2000/rawfile/HEAD/devries_sherman_2000.cellml
		marhl_haberichter_brumen_heinrich_2000/rawfile/HEAD/marhl_haberichter_brumen_heinrich_2000.cellml
37	frog intact muscle	baylor_hollingworth_chandler_2002/rawfile/HEAD/baylor_hollingworth_chandler_2002_f.cellml
38	christopher data-driven computer	baylor_hollingworth_chandler_2002/rawfile/HEAD/baylor_hollingworth_chandler_2002_b.cellml
		baylor_hollingworth_chandler_2002/rawfile/HEAD/baylor_hollingworth_chandler_2002_d.cellml
		luo_rudy_1994/rawfile/HEAD/luo_rudy_1994.cellml
		colegrove_albrecht_friel_2000/rawfile/HEAD/colegrove_albrecht_friel_2000.cellml
		dougherty_wright_yew_2005/rawfile/HEAD/dougherty_wright_yew_2005.cellml
		yamaguchi_takaki_matsubara_yasuhara_suga_1996/rawfile/HEAD/yamaguchi_takaki_matsubara_yasuhara_suga_1996.cellml
		boyett_zhang_garny_holden_2001/rawfile/HEAD/boyett_zhang_garny_holden_2001.cellml
		iribe_kohl_noble_2006/rawfile/HEAD/iribe_kohl_noble_2006.cellml
		izakov_katsnelson_blyakhman_markhasin_shkylar_1991/rawfile/HEAD/izakov_katsnelson_blyakhman_markhasin_shkylar_1991.cellml
		stern_song_sham_yang_boheler_rios_1999/rawfile/HEAD/stern_song_sham_yang_boheler_rios_1999.cellml
		devries_sherman_2000/rawfile/HEAD/devries_sherman_2000.cellml
		marhl_haberichter_brumen_heinrich_2000/rawfile/HEAD/marhl_haberichter_brumen_heinrich_2000.cellml
39	brain model	546/rawfile/HEAD/cloutier_2009.cellml
40	socrates dokos, branko celler, and nigel lovell	baylor_hollingworth_chandler_2002/rawfile/HEAD/baylor_hollingworth_chandler_2002_b.cellml
		baylor_hollingworth_chandler_2002/rawfile/HEAD/baylor_hollingworth_chandler_2002_d.cellml
		luo_rudy_1994/rawfile/HEAD/luo_rudy_1994.cellml
		colegrove_albrecht_friel_2000/rawfile/HEAD/colegrove_albrecht_friel_2000.cellml
		dougherty_wright_yew_2005/rawfile/HEAD/dougherty_wright_yew_2005.cellml
		yamaguchi_takaki_matsubara_yasuhara_suga_1996/rawfile/HEAD/yamaguchi_takaki_matsubara_yasuhara_suga_1996.cellml
		boyett_zhang_garny_holden_2001/rawfile/HEAD/boyett_zhang_garny_holden_2001.cellml
		iribe_kohl_noble_2006/rawfile/HEAD/iribe_kohl_noble_2006.cellml
		izakov_katsnelson_blyakhman_markhasin_shkylar_1991/rawfile/HEAD/izakov_katsnelson_blyakhman_markhasin_shkylar_1991.cellml
		stern_song_sham_yang_boheler_rios_1999/rawfile/HEAD/stern_song_sham_yang_boheler_rios_1999.cellml
		devries_sherman_2000/rawfile/HEAD/devries_sherman_2000.cellml
		marhl_haberichter_brumen_heinrich_2000/rawfile/HEAD/marhl_haberichter_brumen_heinrich_2000.cellml
41	rabbit	baylor_hollingworth_chandler_2002/rawfile/HEAD/baylor_hollingworth_chandler_2002_b.cellml
		baylor_hollingworth_chandler_2002/rawfile/HEAD/baylor_hollingworth_chandler_2002_d.cellml
		luo_rudy_1994/rawfile/HEAD/luo_rudy_1994.cellml
		colegrove_albrecht_friel_2000/rawfile/HEAD/colegrove_albrecht_friel_2000.cellml
		dougherty_wright_yew_2005/rawfile/HEAD/dougherty_wright_yew_2005.cellml
		yamaguchi_takaki_matsubara_yasuhara_suga_1996/rawfile/HEAD/yamaguchi_takaki_matsubara_yasuhara_suga_1996.cellml
		boyett_zhang_garny_holden_2001/rawfile/HEAD/boyett_zhang_garny_holden_2001.cellml
		iribe_kohl_noble_2006/rawfile/HEAD/iribe_kohl_noble_2006.cellml
		izakov_katsnelson_blyakhman_markhasin_shkylar_1991/rawfile/HEAD/izakov_katsnelson_blyakhman_markhasin_shkylar_1991.cellml
		stern_song_sham_yang_boheler_rios_1999/rawfile/HEAD/stern_song_sham_yang_boheler_rios_1999.cellml
		devries_sherman_2000/rawfile/HEAD/devries_sherman_2000.cellml
		marhl_haberichter_brumen_heinrich_2000/rawfile/HEAD/marhl_haberichter_brumen_heinrich_2000.cellml
42	kidney	baylor_hollingworth_chandler_2002/rawfile/HEAD/baylor_hollingworth_chandler_2002_b.cellml
		baylor_hollingworth_chandler_2002/rawfile/HEAD/baylor_hollingworth_chandler_2002_d.cellml
		luo_rudy_1994/rawfile/HEAD/luo_rudy_1994.cellml
		colegrove_albrecht_friel_2000/rawfile/HEAD/colegrove_albrecht_friel_2000.cellml
		267/rawfile/HEAD/mackenzie_1996-mouse-baso.cellml
		267/rawfile/HEAD/SEDML/mackenzie_1996/mackenzie_1996.cellml
		dougherty_wright_yew_2005/rawfile/HEAD/dougherty_wright_yew_2005.cellml
		yamaguchi_takaki_matsubara_yasuhara_suga_1996/rawfile/HEAD/yamaguchi_takaki_matsubara_yasuhara_suga_1996.cellml
		267/rawfile/HEAD/mackenzie_1996.cellml
		boyett_zhang_garny_holden_2001/rawfile/HEAD/boyett_zhang_garny_holden_2001.cellml
		iribe_kohl_noble_2006/rawfile/HEAD/iribe_kohl_noble_2006.cellml
		izakov_katsnelson_blyakhman_markhasin_shkylar_1991/rawfile/HEAD/izakov_katsnelson_blyakhman_markhasin_shkylar_1991.cellml
		stern_song_sham_yang_boheler_rios_1999/rawfile/HEAD/stern_song_sham_yang_boheler_rios_1999.cellml
		devries_sherman_2000/rawfile/HEAD/devries_sherman_2000.cellml
		marhl_haberichter_brumen_heinrich_2000/rawfile/HEAD/marhl_haberichter_brumen_heinrich_2000.cellml
		baylor_hollingworth_chandler_2002/rawfile/HEAD/baylor_hollingworth_chandler_2002_b.cellml

43 protein

		baylor_hollingworth_chandler_2002/rawfile/HEAD/baylor_hollingworth_chandler_2002_d.cellml
		luo_rudy_1994/rawfile/HEAD/luo_rudy_1994.cellml
		colegrove_albrecht_friel_2000/rawfile/HEAD/colegrove_albrecht_friel_2000.cellml
		dougherty_wright_yew_2005/rawfile/HEAD/dougherty_wright_yew_2005.cellml
		yamaguchi_takaki_matsubara_yasuhara_suga_1996/rawfile/HEAD/yamaguchi_takaki_matsubara_yasuhara_suga_1996.cellml
		boyett_zhang_garny_holden_2001/rawfile/HEAD/boyett_zhang_garny_holden_2001.cellml
		iribe_kohl_noble_2006/rawfile/HEAD/iribe_kohl_noble_2006.cellml
		izakov_katsnelson_blyakhman_markhasin_shkylar_1991/rawfile/HEAD/izakov_katsnelson_blyakhman_markhasin_shkylar_1991.cellml
		stern_song_sham_yang_boheler_rios_1999/rawfile/HEAD/stern_song_sham_yang_boheler_rios_1999.cellml
		devries_sherman_2000/rawfile/HEAD/devries_sherman_2000.cellml
		marhl_haberichter_brumen_heinrich_2000/rawfile/HEAD/marhl_haberichter_brumen_heinrich_2000.cellml
44	sodium channel	baylor_hollingworth_chandler_2002/rawfile/HEAD/baylor_hollingworth_chandler_2002_b.cellml
		baylor_hollingworth_chandler_2002/rawfile/HEAD/baylor_hollingworth_chandler_2002_d.cellml
		luo_rudy_1994/rawfile/HEAD/luo_rudy_1994.cellml
		colegrove_albrecht_friel_2000/rawfile/HEAD/colegrove_albrecht_friel_2000.cellml
		dougherty_wright_yew_2005/rawfile/HEAD/dougherty_wright_yew_2005.cellml
		yamaguchi_takaki_matsubara_yasuhara_suga_1996/rawfile/HEAD/yamaguchi_takaki_matsubara_yasuhara_suga_1996.cellml
		boyett_zhang_garny_holden_2001/rawfile/HEAD/boyett_zhang_garny_holden_2001.cellml
		iribe_kohl_noble_2006/rawfile/HEAD/iribe_kohl_noble_2006.cellml
		izakov_katsnelson_blyakhman_markhasin_shkylar_1991/rawfile/HEAD/izakov_katsnelson_blyakhman_markhasin_shkylar_1991.cellml
		stern_song_sham_yang_boheler_rios_1999/rawfile/HEAD/stern_song_sham_yang_boheler_rios_1999.cellml
		devries_sherman_2000/rawfile/HEAD/devries_sherman_2000.cellml
		marhl_haberichter_brumen_heinrich_2000/rawfile/HEAD/marhl_haberichter_brumen_heinrich_2000.cellml
45	mitochondrial modulation of intracellular	fall_keizer_2001/rawfile/HEAD/fall_keizer_2001.cellml
46	a simplified model for mitochondrial atp production	baylor_hollingworth_chandler_2002/rawfile/HEAD/baylor_hollingworth_chandler_2002_b.cellml
		baylor_hollingworth_chandler_2002/rawfile/HEAD/baylor_hollingworth_chandler_2002_d.cellml
		luo_rudy_1994/rawfile/HEAD/luo_rudy_1994.cellml
		colegrove_albrecht_friel_2000/rawfile/HEAD/colegrove_albrecht_friel_2000.cellml
		dougherty_wright_yew_2005/rawfile/HEAD/dougherty_wright_yew_2005.cellml
		yamaguchi_takaki_matsubara_yasuhara_suga_1996/rawfile/HEAD/yamaguchi_takaki_matsubara_yasuhara_suga_1996.cellml
		boyett_zhang_garny_holden_2001/rawfile/HEAD/boyett_zhang_garny_holden_2001.cellml
		iribe_kohl_noble_2006/rawfile/HEAD/iribe_kohl_noble_2006.cellml
		izakov_katsnelson_blyakhman_markhasin_shkylar_1991/rawfile/HEAD/izakov_katsnelson_blyakhman_markhasin_shkylar_1991.cellml
		stern_song_sham_yang_boheler_rios_1999/rawfile/HEAD/stern_song_sham_yang_boheler_rios_1999.cellml
		devries_sherman_2000/rawfile/HEAD/devries_sherman_2000.cellml
		marhl_haberichter_brumen_heinrich_2000/rawfile/HEAD/marhl_haberichter_brumen_heinrich_2000.cellml
47	an integrated model of cardiac mitochondrial energy metabolism and calcium dynamics	cortassa_aon_marban_winslow_orourke_2003/rawfile/HEAD/cortassa_aon_marban_winslow_orourke_2003.cellml
48	complex calcium oscillations and the role of mitochondria and cytosolic proteins	baylor_hollingworth_chandler_2002/rawfile/HEAD/baylor_hollingworth_chandler_2002_b.cellml
		baylor_hollingworth_chandler_2002/rawfile/HEAD/baylor_hollingworth_chandler_2002_d.cellml
		luo_rudy_1994/rawfile/HEAD/luo_rudy_1994.cellml
		colegrove_albrecht_friel_2000/rawfile/HEAD/colegrove_albrecht_friel_2000.cellml
		dougherty_wright_yew_2005/rawfile/HEAD/dougherty_wright_yew_2005.cellml
		yamaguchi_takaki_matsubara_yasuhara_suga_1996/rawfile/HEAD/yamaguchi_takaki_matsubara_yasuhara_suga_1996.cellml
		boyett_zhang_garny_holden_2001/rawfile/HEAD/boyett_zhang_garny_holden_2001.cellml
		iribe_kohl_noble_2006/rawfile/HEAD/iribe_kohl_noble_2006.cellml
		izakov_katsnelson_blyakhman_markhasin_shkylar_1991/rawfile/HEAD/izakov_katsnelson_blyakhman_markhasin_shkylar_1991.cellml
		stern_song_sham_yang_boheler_rios_1999/rawfile/HEAD/stern_song_sham_yang_boheler_rios_1999.cellml
		devries_sherman_2000/rawfile/HEAD/devries_sherman_2000.cellml
		marhl_haberichter_brumen_heinrich_2000/rawfile/HEAD/marhl_haberichter_brumen_heinrich_2000.cellml
49	a mitochondrial oscillator dependent on reactive oxygen species	cortassa_aon_marban_winslow_orourke_2003/rawfile/HEAD/cortassa_aon_marban_winslow_orourke_2003.cellml
50	computer modeling of mitochondrial tricarboxylic acid cycle, oxidative phosphorylation, metabolite transport, and electrophysiology	baylor_hollingworth_chandler_2002/rawfile/HEAD/baylor_hollingworth_chandler_2002_b.cellml
		baylor_hollingworth_chandler_2002/rawfile/HEAD/baylor_hollingworth_chandler_2002_d.cellml
		luo_rudy_1994/rawfile/HEAD/luo_rudy_1994.cellml
		colegrove_albrecht_friel_2000/rawfile/HEAD/colegrove_albrecht_friel_2000.cellml
		dougherty_wright_yew_2005/rawfile/HEAD/dougherty_wright_yew_2005.cellml
		yamaguchi_takaki_matsubara_yasuhara_suga_1996/rawfile/HEAD/yamaguchi_takaki_matsubara_yasuhara_suga_1996.cellml
		boyett_zhang_garny_holden_2001/rawfile/HEAD/boyett_zhang_garny_holden_2001.cellml
		iribe_kohl_noble_2006/rawfile/HEAD/iribe_kohl_noble_2006.cellml

		izakov_katsnelson_blyakhman_markhasin_shkylar_1991/rawfile/HEAD/izakov_katsnelson_blyakhman_markhasin_shkylar_1991.cellml
		stern_song_sham_yang_boheler_rios_1999/rawfile/HEAD/stern_song_sham_yang_boheler_rios_1999.cellml
		devries_sherman_2000/rawfile/HEAD/devries_sherman_2000.cellml
		marhl_haberichter_brumen_heinrich_2000/rawfile/HEAD/marhl_haberichter_brumen_heinrich_2000.cellml
51	mitochondria	baylor_hollingworth_chandler_2002/rawfile/HEAD/baylor_hollingworth_chandler_2002_b.cellml
		baylor_hollingworth_chandler_2002/rawfile/HEAD/baylor_hollingworth_chandler_2002_d.cellml
		luo_rudy_1994/rawfile/HEAD/luo_rudy_1994.cellml
		colegrove_albrecht_friel_2000/rawfile/HEAD/colegrove_albrecht_friel_2000.cellml
		cortassa_aon_marban_winslow_orourke_2003/rawfile/HEAD/cortassa_aon_marban_winslow_orourke_2003.cellml
		dougherty_wright_yew_2005/rawfile/HEAD/dougherty_wright_yew_2005.cellml
		yamaguchi_takaki_matsubara_yasuhara_suga_1996/rawfile/HEAD/yamaguchi_takaki_matsubara_yasuhara_suga_1996.cellml
		boyett_zhang_garny_holden_2001/rawfile/HEAD/boyett_zhang_garny_holden_2001.cellml
		fall_keizer_2001/rawfile/HEAD/fall_keizer_2001.cellml
		iribe_kohl_noble_2006/rawfile/HEAD/iribe_kohl_noble_2006.cellml
		546/rawfile/HEAD/cloutier_2009.cellml
		albrecht_colegrove_friel_2002/rawfile/HEAD/albrecht_colegrove_friel_2002.cellml
		izakov_katsnelson_blyakhman_markhasin_shkylar_1991/rawfile/HEAD/izakov_katsnelson_blyakhman_markhasin_shkylar_1991.cellml
		stern_song_sham_yang_boheler_rios_1999/rawfile/HEAD/stern_song_sham_yang_boheler_rios_1999.cellml
		devries_sherman_2000/rawfile/HEAD/devries_sherman_2000.cellml
		marhl_haberichter_brumen_heinrich_2000/rawfile/HEAD/marhl_haberichter_brumen_heinrich_2000.cellml
52	t cell receptors	baylor_hollingworth_chandler_2002/rawfile/HEAD/baylor_hollingworth_chandler_2002_b.cellml
		baylor_hollingworth_chandler_2002/rawfile/HEAD/baylor_hollingworth_chandler_2002_d.cellml
		luo_rudy_1994/rawfile/HEAD/luo_rudy_1994.cellml
		colegrove_albrecht_friel_2000/rawfile/HEAD/colegrove_albrecht_friel_2000.cellml
		dougherty_wright_yew_2005/rawfile/HEAD/dougherty_wright_yew_2005.cellml
		yamaguchi_takaki_matsubara_yasuhara_suga_1996/rawfile/HEAD/yamaguchi_takaki_matsubara_yasuhara_suga_1996.cellml
		boyett_zhang_garny_holden_2001/rawfile/HEAD/boyett_zhang_garny_holden_2001.cellml
		iribe_kohl_noble_2006/rawfile/HEAD/iribe_kohl_noble_2006.cellml
		izakov_katsnelson_blyakhman_markhasin_shkylar_1991/rawfile/HEAD/izakov_katsnelson_blyakhman_markhasin_shkylar_1991.cellml
		stern_song_sham_yang_boheler_rios_1999/rawfile/HEAD/stern_song_sham_yang_boheler_rios_1999.cellml
		devries_sherman_2000/rawfile/HEAD/devries_sherman_2000.cellml
		marhl_haberichter_brumen_heinrich_2000/rawfile/HEAD/marhl_haberichter_brumen_heinrich_2000.cellml
53	respiratory	baylor_hollingworth_chandler_2002/rawfile/HEAD/baylor_hollingworth_chandler_2002_b.cellml
		baylor_hollingworth_chandler_2002/rawfile/HEAD/baylor_hollingworth_chandler_2002_d.cellml
		luo_rudy_1994/rawfile/HEAD/luo_rudy_1994.cellml
		colegrove_albrecht_friel_2000/rawfile/HEAD/colegrove_albrecht_friel_2000.cellml
		dougherty_wright_yew_2005/rawfile/HEAD/dougherty_wright_yew_2005.cellml
		yamaguchi_takaki_matsubara_yasuhara_suga_1996/rawfile/HEAD/yamaguchi_takaki_matsubara_yasuhara_suga_1996.cellml
		boyett_zhang_garny_holden_2001/rawfile/HEAD/boyett_zhang_garny_holden_2001.cellml
		iribe_kohl_noble_2006/rawfile/HEAD/iribe_kohl_noble_2006.cellml
		izakov_katsnelson_blyakhman_markhasin_shkylar_1991/rawfile/HEAD/izakov_katsnelson_blyakhman_markhasin_shkylar_1991.cellml
		stern_song_sham_yang_boheler_rios_1999/rawfile/HEAD/stern_song_sham_yang_boheler_rios_1999.cellml
		devries_sherman_2000/rawfile/HEAD/devries_sherman_2000.cellml
		marhl_haberichter_brumen_heinrich_2000/rawfile/HEAD/marhl_haberichter_brumen_heinrich_2000.cellml
54	atrial ap	baylor_hollingworth_chandler_2002/rawfile/HEAD/baylor_hollingworth_chandler_2002_b.cellml
		baylor_hollingworth_chandler_2002/rawfile/HEAD/baylor_hollingworth_chandler_2002_d.cellml
		luo_rudy_1994/rawfile/HEAD/luo_rudy_1994.cellml
		colegrove_albrecht_friel_2000/rawfile/HEAD/colegrove_albrecht_friel_2000.cellml
		dougherty_wright_yew_2005/rawfile/HEAD/dougherty_wright_yew_2005.cellml
		yamaguchi_takaki_matsubara_yasuhara_suga_1996/rawfile/HEAD/yamaguchi_takaki_matsubara_yasuhara_suga_1996.cellml
		boyett_zhang_garny_holden_2001/rawfile/HEAD/boyett_zhang_garny_holden_2001.cellml
		iribe_kohl_noble_2006/rawfile/HEAD/iribe_kohl_noble_2006.cellml
		izakov_katsnelson_blyakhman_markhasin_shkylar_1991/rawfile/HEAD/izakov_katsnelson_blyakhman_markhasin_shkylar_1991.cellml
		stern_song_sham_yang_boheler_rios_1999/rawfile/HEAD/stern_song_sham_yang_boheler_rios_1999.cellml
		devries_sherman_2000/rawfile/HEAD/devries_sherman_2000.cellml
		marhl_haberichter_brumen_heinrich_2000/rawfile/HEAD/marhl_haberichter_brumen_heinrich_2000.cellml
		baylor_hollingworth_chandler_2002/rawfile/HEAD/baylor_hollingworth_chandler_2002_b.cellml
		baylor_hollingworth_chandler_2002/rawfile/HEAD/baylor_hollingworth_chandler_2002_d.cellml
		luo_rudy_1994/rawfile/HEAD/luo_rudy_1994.cellml
		colegrove_albrecht_friel_2000/rawfile/HEAD/colegrove_albrecht_friel_2000.cellml

55 calcium dynamics

		cortassa_aon_marban_winslow_orourke_2003/rawfile/HEAD/cortassa_aon_marban_winslow_orourke_2003.cellml
		dougherty_wright_yew_2005/rawfile/HEAD/dougherty_wright_yew_2005.cellml
		yamaguchi_takaki_matsubara_yasuhara_suga_1996/rawfile/HEAD/yamaguchi_takaki_matsubara_yasuhara_suga_1996.cellml
		noble_noble_2001/rawfile/HEAD/noble_noble_2001.cellml
		boyett_zhang_garny_holden_2001/rawfile/HEAD/boyett_zhang_garny_holden_2001.cellml
		iribe_kohl_noble_2006/rawfile/HEAD/iribe_kohl_noble_2006.cellml
		shannon_wang_puglisi_weber_bers_2004/rawfile/HEAD/shannon_wang_puglisi_weber_bers_2004_a.cellml
		izakov_katsnelson_blyakhman_markhasin_shkylar_1991/rawfile/HEAD/izakov_katsnelson_blyakhman_markhasin_shkylar_1991.cellml
		stern_song_sham_yang_boheler_rios_1999/rawfile/HEAD/stern_song_sham_yang_boheler_rios_1999.cellml
		shiferaw_watanabe_garfinkel_weiss_karma_2003/rawfile/HEAD/shiferaw_watanabe_garfinkel_weiss_karma_2003.cellml
		shannon_wang_puglisi_weber_bers_2004/rawfile/HEAD/shannon_wang_puglisi_weber_bers_2004_b.cellml
		devries_sherman_2000/rawfile/HEAD/devries_sherman_2000.cellml
		marhl_haberichter_brumen_heinrich_2000/rawfile/HEAD/marhl_haberichter_brumen_heinrich_2000.cellml
56	luo rudy phase -ii model	baylor_hollingworth_chandler_2002/rawfile/HEAD/baylor_hollingworth_chandler_2002_b.cellml
		baylor_hollingworth_chandler_2002/rawfile/HEAD/baylor_hollingworth_chandler_2002_d.cellml
		luo_rudy_1994/rawfile/HEAD/luo_rudy_1994.cellml
		colegrove_albrecht_friel_2000/rawfile/HEAD/colegrove_albrecht_friel_2000.cellml
		dougherty_wright_yew_2005/rawfile/HEAD/dougherty_wright_yew_2005.cellml
		yamaguchi_takaki_matsubara_yasuhara_suga_1996/rawfile/HEAD/yamaguchi_takaki_matsubara_yasuhara_suga_1996.cellml
		boyett_zhang_garny_holden_2001/rawfile/HEAD/boyett_zhang_garny_holden_2001.cellml
		iribe_kohl_noble_2006/rawfile/HEAD/iribe_kohl_noble_2006.cellml
		izakov_katsnelson_blyakhman_markhasin_shkylar_1991/rawfile/HEAD/izakov_katsnelson_blyakhman_markhasin_shkylar_1991.cellml
		stern_song_sham_yang_boheler_rios_1999/rawfile/HEAD/stern_song_sham_yang_boheler_rios_1999.cellml
		devries_sherman_2000/rawfile/HEAD/devries_sherman_2000.cellml
		marhl_haberichter_brumen_heinrich_2000/rawfile/HEAD/marhl_haberichter_brumen_heinrich_2000.cellml
57	magnus, keizer	fall_keizer_2001/rawfile/HEAD/fall_keizer_2001.cellml
58	purkinje cell model	baylor_hollingworth_chandler_2002/rawfile/HEAD/baylor_hollingworth_chandler_2002_b.cellml
		baylor_hollingworth_chandler_2002/rawfile/HEAD/baylor_hollingworth_chandler_2002_d.cellml
		luo_rudy_1994/rawfile/HEAD/luo_rudy_1994.cellml
		colegrove_albrecht_friel_2000/rawfile/HEAD/colegrove_albrecht_friel_2000.cellml
		dougherty_wright_yew_2005/rawfile/HEAD/dougherty_wright_yew_2005.cellml
		yamaguchi_takaki_matsubara_yasuhara_suga_1996/rawfile/HEAD/yamaguchi_takaki_matsubara_yasuhara_suga_1996.cellml
		boyett_zhang_garny_holden_2001/rawfile/HEAD/boyett_zhang_garny_holden_2001.cellml
		iribe_kohl_noble_2006/rawfile/HEAD/iribe_kohl_noble_2006.cellml
		izakov_katsnelson_blyakhman_markhasin_shkylar_1991/rawfile/HEAD/izakov_katsnelson_blyakhman_markhasin_shkylar_1991.cellml
		stern_song_sham_yang_boheler_rios_1999/rawfile/HEAD/stern_song_sham_yang_boheler_rios_1999.cellml
		devries_sherman_2000/rawfile/HEAD/devries_sherman_2000.cellml
		marhl_haberichter_brumen_heinrich_2000/rawfile/HEAD/marhl_haberichter_brumen_heinrich_2000.cellml
59	human atrial cell model	baylor_hollingworth_chandler_2002/rawfile/HEAD/baylor_hollingworth_chandler_2002_b.cellml
		baylor_hollingworth_chandler_2002/rawfile/HEAD/baylor_hollingworth_chandler_2002_d.cellml
		luo_rudy_1994/rawfile/HEAD/luo_rudy_1994.cellml
		colegrove_albrecht_friel_2000/rawfile/HEAD/colegrove_albrecht_friel_2000.cellml
		dougherty_wright_yew_2005/rawfile/HEAD/dougherty_wright_yew_2005.cellml
		yamaguchi_takaki_matsubara_yasuhara_suga_1996/rawfile/HEAD/yamaguchi_takaki_matsubara_yasuhara_suga_1996.cellml
		boyett_zhang_garny_holden_2001/rawfile/HEAD/boyett_zhang_garny_holden_2001.cellml
		iribe_kohl_noble_2006/rawfile/HEAD/iribe_kohl_noble_2006.cellml
		izakov_katsnelson_blyakhman_markhasin_shkylar_1991/rawfile/HEAD/izakov_katsnelson_blyakhman_markhasin_shkylar_1991.cellml
		stern_song_sham_yang_boheler_rios_1999/rawfile/HEAD/stern_song_sham_yang_boheler_rios_1999.cellml
		devries_sherman_2000/rawfile/HEAD/devries_sherman_2000.cellml
		marhl_haberichter_brumen_heinrich_2000/rawfile/HEAD/marhl_haberichter_brumen_heinrich_2000.cellml
60	purkinje fibre human	baylor_hollingworth_chandler_2002/rawfile/HEAD/baylor_hollingworth_chandler_2002_b.cellml
		baylor_hollingworth_chandler_2002/rawfile/HEAD/baylor_hollingworth_chandler_2002_d.cellml
		luo_rudy_1994/rawfile/HEAD/luo_rudy_1994.cellml
		colegrove_albrecht_friel_2000/rawfile/HEAD/colegrove_albrecht_friel_2000.cellml
		dougherty_wright_yew_2005/rawfile/HEAD/dougherty_wright_yew_2005.cellml
		yamaguchi_takaki_matsubara_yasuhara_suga_1996/rawfile/HEAD/yamaguchi_takaki_matsubara_yasuhara_suga_1996.cellml
		boyett_zhang_garny_holden_2001/rawfile/HEAD/boyett_zhang_garny_holden_2001.cellml
		iribe_kohl_noble_2006/rawfile/HEAD/iribe_kohl_noble_2006.cellml
		izakov_katsnelson_blyakhman_markhasin_shkylar_1991/rawfile/HEAD/izakov_katsnelson_blyakhman_markhasin_shkylar_1991.cellml
		stern_song_sham_yang_boheler_rios_1999/rawfile/HEAD/stern_song_sham_yang_boheler_rios_1999.cellml

		devries_sherman_2000/rawfile/HEAD/devries_sherman_2000.cellml
		marhl_haberichter_brumen_heinrich_2000/rawfile/HEAD/marhl_haberichter_brumen_heinrich_2000.cellml
61	human purkinje	baylor_hollingworth_chandler_2002/rawfile/HEAD/baylor_hollingworth_chandler_2002_b.cellml
		baylor_hollingworth_chandler_2002/rawfile/HEAD/baylor_hollingworth_chandler_2002_d.cellml
		luo_rudy_1994/rawfile/HEAD/luo_rudy_1994.cellml
		colegrove_albrecht_friel_2000/rawfile/HEAD/colegrove_albrecht_friel_2000.cellml
		dougherty_wright_yew_2005/rawfile/HEAD/dougherty_wright_yew_2005.cellml
		yamaguchi_takaki_matsubara_yasuhara_suga_1996/rawfile/HEAD/yamaguchi_takaki_matsubara_yasuhara_suga_1996.cellml
		boyett_zhang_garny_holden_2001/rawfile/HEAD/boyett_zhang_garny_holden_2001.cellml
		iribe_kohl_noble_2006/rawfile/HEAD/iribe_kohl_noble_2006.cellml
		izakov_katsnelson_blyakhman_markhasin_shkylar_1991/rawfile/HEAD/izakov_katsnelson_blyakhman_markhasin_shkylar_1991.cellml
		stern_song_sham_yang_boheler_rios_1999/rawfile/HEAD/stern_song_sham_yang_boheler_rios_1999.cellml
		devries_sherman_2000/rawfile/HEAD/devries_sherman_2000.cellml
		marhl_haberichter_brumen_heinrich_2000/rawfile/HEAD/marhl_haberichter_brumen_heinrich_2000.cellml
62	matlab calcium	faber_rudy_2000/rawfile/HEAD/faber_rudy_modified_version_2000.cellml
63	an integrative dynamic model of brain energy metabolism using in vivo neurochemical measurements	546/rawfile/HEAD/cloutier_2009.cellml
64	ryanodine	baylor_hollingworth_chandler_2002/rawfile/HEAD/baylor_hollingworth_chandler_2002_b.cellml
		baylor_hollingworth_chandler_2002/rawfile/HEAD/baylor_hollingworth_chandler_2002_d.cellml
		luo_rudy_1994/rawfile/HEAD/luo_rudy_1994.cellml
		michailova_mcculloch_2001/rawfile/HEAD/michailova_mcculloch_2001.cellml
		colegrove_albrecht_friel_2000/rawfile/HEAD/colegrove_albrecht_friel_2000.cellml
		dougherty_wright_yew_2005/rawfile/HEAD/dougherty_wright_yew_2005.cellml
		jafri_rice_winslow_1998/rawfile/HEAD/jafri_rice_winslow_1998_a.cellml
		jafri_rice_winslow_1998/rawfile/HEAD/jafri_rice_winslow_1998_b.cellml
		yamaguchi_takaki_matsubara_yasuhara_suga_1996/rawfile/HEAD/yamaguchi_takaki_matsubara_yasuhara_suga_1996.cellml
		boyett_zhang_garny_holden_2001/rawfile/HEAD/boyett_zhang_garny_holden_2001.cellml
		iribe_kohl_noble_2006/rawfile/HEAD/iribe_kohl_noble_2006.cellml
		winslow_rice_jafri_marban_ororke_1999/rawfile/HEAD/winslow_rice_jafri_marban_ororke_1999.cellml
		izakov_katsnelson_blyakhman_markhasin_shkylar_1991/rawfile/HEAD/izakov_katsnelson_blyakhman_markhasin_shkylar_1991.cellml
		stern_song_sham_yang_boheler_rios_1999/rawfile/HEAD/stern_song_sham_yang_boheler_rios_1999.cellml
		devries_sherman_2000/rawfile/HEAD/devries_sherman_2000.cellml
		marhl_haberichter_brumen_heinrich_2000/rawfile/HEAD/marhl_haberichter_brumen_heinrich_2000.cellml
65	magnus, keizer, 1998	magnus_keizer_1998/rawfile/HEAD/magnus_keizer_1998.cellml
		fall_keizer_2001/rawfile/HEAD/fall_keizer_2001.cellml
66	brain	546/rawfile/HEAD/cloutier_2009.cellml
67	noble'98	noble_noble_2001/rawfile/HEAD/noble_noble_2001.cellml
68	nuclear erythyroid 2	baylor_hollingworth_chandler_2002/rawfile/HEAD/baylor_hollingworth_chandler_2002_b.cellml
		baylor_hollingworth_chandler_2002/rawfile/HEAD/baylor_hollingworth_chandler_2002_d.cellml
		luo_rudy_1994/rawfile/HEAD/luo_rudy_1994.cellml
		colegrove_albrecht_friel_2000/rawfile/HEAD/colegrove_albrecht_friel_2000.cellml
		dougherty_wright_yew_2005/rawfile/HEAD/dougherty_wright_yew_2005.cellml
		yamaguchi_takaki_matsubara_yasuhara_suga_1996/rawfile/HEAD/yamaguchi_takaki_matsubara_yasuhara_suga_1996.cellml
		boyett_zhang_garny_holden_2001/rawfile/HEAD/boyett_zhang_garny_holden_2001.cellml
		iribe_kohl_noble_2006/rawfile/HEAD/iribe_kohl_noble_2006.cellml
		izakov_katsnelson_blyakhman_markhasin_shkylar_1991/rawfile/HEAD/izakov_katsnelson_blyakhman_markhasin_shkylar_1991.cellml
		stern_song_sham_yang_boheler_rios_1999/rawfile/HEAD/stern_song_sham_yang_boheler_rios_1999.cellml
		devries_sherman_2000/rawfile/HEAD/devries_sherman_2000.cellml
		marhl_haberichter_brumen_heinrich_2000/rawfile/HEAD/marhl_haberichter_brumen_heinrich_2000.cellml
69	cytosolic inhibitor	baylor_hollingworth_chandler_2002/rawfile/HEAD/baylor_hollingworth_chandler_2002_b.cellml
		baylor_hollingworth_chandler_2002/rawfile/HEAD/baylor_hollingworth_chandler_2002_d.cellml
		267/rawfile/HEAD/eskandari_2005.cellml
		luo_rudy_1994/rawfile/HEAD/luo_rudy_1994.cellml
		colegrove_albrecht_friel_2000/rawfile/HEAD/colegrove_albrecht_friel_2000.cellml
		dougherty_wright_yew_2005/rawfile/HEAD/dougherty_wright_yew_2005.cellml
		yamaguchi_takaki_matsubara_yasuhara_suga_1996/rawfile/HEAD/yamaguchi_takaki_matsubara_yasuhara_suga_1996.cellml
		boyett_zhang_garny_holden_2001/rawfile/HEAD/boyett_zhang_garny_holden_2001.cellml
		267/rawfile/HEAD/SEDML/eskandari_2005/eskandari_2005.cellml
		iribe_kohl_noble_2006/rawfile/HEAD/iribe_kohl_noble_2006.cellml

		izakov_katsnelson_blyakhman_markhasin_shkylar_1991/rawfile/HEAD/izakov_katsnelson_blyakhman_markhasin_shkylar_1991.cellml
		stern_song_sham_yang_boheler_rios_1999/rawfile/HEAD/stern_song_sham_yang_boheler_rios_1999.cellml
		devries_sherman_2000/rawfile/HEAD/devries_sherman_2000.cellml
		marhl_haberichter_brumen_heinrich_2000/rawfile/HEAD/marhl_haberichter_brumen_heinrich_2000.cellml
70	lumped-parameter cardiovascular	baylor_hollingworth_chandler_2002/rawfile/HEAD/baylor_hollingworth_chandler_2002_b.cellml
		baylor_hollingworth_chandler_2002/rawfile/HEAD/baylor_hollingworth_chandler_2002_d.cellml
		luo_rudy_1994/rawfile/HEAD/luo_rudy_1994.cellml
		colegrove_albrecht_friel_2000/rawfile/HEAD/colegrove_albrecht_friel_2000.cellml
		dougherty_wright_yew_2005/rawfile/HEAD/dougherty_wright_yew_2005.cellml
		yamaguchi_takaki_matsubara_yasuhara_suga_1996/rawfile/HEAD/yamaguchi_takaki_matsubara_yasuhara_suga_1996.cellml
		boyett_zhang_garny_holden_2001/rawfile/HEAD/boyett_zhang_garny_holden_2001.cellml
		iribe_kohl_noble_2006/rawfile/HEAD/iribe_kohl_noble_2006.cellml
		izakov_katsnelson_blyakhman_markhasin_shkylar_1991/rawfile/HEAD/izakov_katsnelson_blyakhman_markhasin_shkylar_1991.cellml
		stern_song_sham_yang_boheler_rios_1999/rawfile/HEAD/stern_song_sham_yang_boheler_rios_1999.cellml
		devries_sherman_2000/rawfile/HEAD/devries_sherman_2000.cellml
		marhl_haberichter_brumen_heinrich_2000/rawfile/HEAD/marhl_haberichter_brumen_heinrich_2000.cellml
71	mackenzie, loo, panayotova-heiermann and wright	267/rawfile/HEAD/mackenzie_1996-mouse-baso.cellml
		267/rawfile/HEAD/SEDML/mackenzie_1996/mackenzie_1996.cellml
		267/rawfile/HEAD/mackenzie_1996.cellml
72	calcium cerebral palsy	bertram_satin_zhang_smolen_sherman_2004/rawfile/HEAD/bertram_satin_zhang_smolen_sherman_2004_b.cellml
		baylor_hollingworth_chandler_2002/rawfile/HEAD/baylor_hollingworth_chandler_2002_b.cellml
		baylor_hollingworth_chandler_2002/rawfile/HEAD/baylor_hollingworth_chandler_2002_d.cellml
		luo_rudy_1994/rawfile/HEAD/luo_rudy_1994.cellml
		colegrove_albrecht_friel_2000/rawfile/HEAD/colegrove_albrecht_friel_2000.cellml
		dougherty_wright_yew_2005/rawfile/HEAD/dougherty_wright_yew_2005.cellml
		yamaguchi_takaki_matsubara_yasuhara_suga_1996/rawfile/HEAD/yamaguchi_takaki_matsubara_yasuhara_suga_1996.cellml
		boyett_zhang_garny_holden_2001/rawfile/HEAD/boyett_zhang_garny_holden_2001.cellml
		267/rawfile/HEAD/bindschadler_sneyd_2001.cellml
		iribe_kohl_noble_2006/rawfile/HEAD/iribe_kohl_noble_2006.cellml
		magnus_keizer_1998/rawfile/HEAD/magnus_keizer_1998.cellml
		bertram_satin_zhang_smolen_sherman_2004/rawfile/HEAD/bertram_satin_zhang_smolen_sherman_2004_a.cellml
		izakov_katsnelson_blyakhman_markhasin_shkylar_1991/rawfile/HEAD/izakov_katsnelson_blyakhman_markhasin_shkylar_1991.cellml
		stern_song_sham_yang_boheler_rios_1999/rawfile/HEAD/stern_song_sham_yang_boheler_rios_1999.cellml
		bindschadler_sneyd_2001/rawfile/HEAD/bindschadler_sneyd_2001.cellml
		devries_sherman_2000/rawfile/HEAD/devries_sherman_2000.cellml
		marhl_haberichter_brumen_heinrich_2000/rawfile/HEAD/marhl_haberichter_brumen_heinrich_2000.cellml
73	ventricular	baylor_hollingworth_chandler_2002/rawfile/HEAD/baylor_hollingworth_chandler_2002_b.cellml
		baylor_hollingworth_chandler_2002/rawfile/HEAD/baylor_hollingworth_chandler_2002_d.cellml
		luo_rudy_1994/rawfile/HEAD/luo_rudy_1994.cellml
		colegrove_albrecht_friel_2000/rawfile/HEAD/colegrove_albrecht_friel_2000.cellml
		dougherty_wright_yew_2005/rawfile/HEAD/dougherty_wright_yew_2005.cellml
		yamaguchi_takaki_matsubara_yasuhara_suga_1996/rawfile/HEAD/yamaguchi_takaki_matsubara_yasuhara_suga_1996.cellml
		boyett_zhang_garny_holden_2001/rawfile/HEAD/boyett_zhang_garny_holden_2001.cellml
		iribe_kohl_noble_2006/rawfile/HEAD/iribe_kohl_noble_2006.cellml
		izakov_katsnelson_blyakhman_markhasin_shkylar_1991/rawfile/HEAD/izakov_katsnelson_blyakhman_markhasin_shkylar_1991.cellml
		stern_song_sham_yang_boheler_rios_1999/rawfile/HEAD/stern_song_sham_yang_boheler_rios_1999.cellml
		shiferaw_watanabe_garfinkel_weiss_karma_2003/rawfile/HEAD/shiferaw_watanabe_garfinkel_weiss_karma_2003.cellml
		devries_sherman_2000/rawfile/HEAD/devries_sherman_2000.cellml
		marhl_haberichter_brumen_heinrich_2000/rawfile/HEAD/marhl_haberichter_brumen_heinrich_2000.cellml
74	calcium heart	michailova_mcculloch_2001/rawfile/HEAD/michailova_mcculloch_2001.cellml
		jafri_rice_winslow_1998/rawfile/HEAD/jafri_rice_winslow_1998_a.cellml
		winslow_rice_jafri_marban_ororke_1999/rawfile/HEAD/winslow_rice_jafri_marban_ororke_1999.cellml
		jafri_rice_winslow_1998/rawfile/HEAD/jafri_rice_winslow_1998_b.cellml
75	noble ventricle 2000	tentusscher_noble_noble_panfilov_2004/rawfile/HEAD/tentusscher_noble_noble_panfilov_2004_c.cellml
		tentusscher_noble_noble_panfilov_2004/rawfile/HEAD/tentusscher_noble_noble_panfilov_2004_b.cellml
		tentusscher_noble_noble_panfilov_2004/rawfile/HEAD/tentusscher_noble_noble_panfilov_2004_a.cellml
		baylor_hollingworth_chandler_2002/rawfile/HEAD/baylor_hollingworth_chandler_2002_b.cellml
		baylor_hollingworth_chandler_2002/rawfile/HEAD/baylor_hollingworth_chandler_2002_d.cellml
		luo_rudy_1994/rawfile/HEAD/luo_rudy_1994.cellml
76	mapk human	

		colegrove_albrecht_friel_2000/rawfile/HEAD/colegrove_albrecht_friel_2000.cellml
		dougherty_wright_yew_2005/rawfile/HEAD/dougherty_wright_yew_2005.cellml
		yamaguchi_takaki_matsubara_yasuhara_suga_1996/rawfile/HEAD/yamaguchi_takaki_matsubara_yasuhara_suga_1996.cellml
		boyett_zhang_garny_holden_2001/rawfile/HEAD/boyett_zhang_garny_holden_2001.cellml
		iribe_kohl_noble_2006/rawfile/HEAD/iribe_kohl_noble_2006.cellml
		izakov_katsnelson_blyakhman_markhasin_shkylar_1991/rawfile/HEAD/izakov_katsnelson_blyakhman_markhasin_shkylar_1991.cellml
		stern_song_sham_yang_boheler_rios_1999/rawfile/HEAD/stern_song_sham_yang_boheler_rios_1999.cellml
		devries_sherman_2000/rawfile/HEAD/devries_sherman_2000.cellml
		marhl_haberichter_brumen_heinrich_2000/rawfile/HEAD/marhl_haberichter_brumen_heinrich_2000.cellml
77	mapk	baylor_hollingworth_chandler_2002/rawfile/HEAD/baylor_hollingworth_chandler_2002_b.cellml
		baylor_hollingworth_chandler_2002/rawfile/HEAD/baylor_hollingworth_chandler_2002_d.cellml
		luo_rudy_1994/rawfile/HEAD/luo_rudy_1994.cellml
		colegrove_albrecht_friel_2000/rawfile/HEAD/colegrove_albrecht_friel_2000.cellml
		dougherty_wright_yew_2005/rawfile/HEAD/dougherty_wright_yew_2005.cellml
		yamaguchi_takaki_matsubara_yasuhara_suga_1996/rawfile/HEAD/yamaguchi_takaki_matsubara_yasuhara_suga_1996.cellml
		boyett_zhang_garny_holden_2001/rawfile/HEAD/boyett_zhang_garny_holden_2001.cellml
		iribe_kohl_noble_2006/rawfile/HEAD/iribe_kohl_noble_2006.cellml
		izakov_katsnelson_blyakhman_markhasin_shkylar_1991/rawfile/HEAD/izakov_katsnelson_blyakhman_markhasin_shkylar_1991.cellml
		stern_song_sham_yang_boheler_rios_1999/rawfile/HEAD/stern_song_sham_yang_boheler_rios_1999.cellml
		devries_sherman_2000/rawfile/HEAD/devries_sherman_2000.cellml
		marhl_haberichter_brumen_heinrich_2000/rawfile/HEAD/marhl_haberichter_brumen_heinrich_2000.cellml
78	electrolytes disturbence with complete heart block	baylor_hollingworth_chandler_2002/rawfile/HEAD/baylor_hollingworth_chandler_2002_b.cellml
		baylor_hollingworth_chandler_2002/rawfile/HEAD/baylor_hollingworth_chandler_2002_d.cellml
		luo_rudy_1994/rawfile/HEAD/luo_rudy_1994.cellml
		colegrove_albrecht_friel_2000/rawfile/HEAD/colegrove_albrecht_friel_2000.cellml
		dougherty_wright_yew_2005/rawfile/HEAD/dougherty_wright_yew_2005.cellml
		yamaguchi_takaki_matsubara_yasuhara_suga_1996/rawfile/HEAD/yamaguchi_takaki_matsubara_yasuhara_suga_1996.cellml
		boyett_zhang_garny_holden_2001/rawfile/HEAD/boyett_zhang_garny_holden_2001.cellml
		iribe_kohl_noble_2006/rawfile/HEAD/iribe_kohl_noble_2006.cellml
		izakov_katsnelson_blyakhman_markhasin_shkylar_1991/rawfile/HEAD/izakov_katsnelson_blyakhman_markhasin_shkylar_1991.cellml
		stern_song_sham_yang_boheler_rios_1999/rawfile/HEAD/stern_song_sham_yang_boheler_rios_1999.cellml
		devries_sherman_2000/rawfile/HEAD/devries_sherman_2000.cellml
		marhl_haberichter_brumen_heinrich_2000/rawfile/HEAD/marhl_haberichter_brumen_heinrich_2000.cellml
79	mitochondrial	fall_keizer_2001/rawfile/HEAD/fall_keizer_2001.cellml
80	atp	baylor_hollingworth_chandler_2002/rawfile/HEAD/baylor_hollingworth_chandler_2002_b.cellml
		baylor_hollingworth_chandler_2002/rawfile/HEAD/baylor_hollingworth_chandler_2002_d.cellml
		luo_rudy_1994/rawfile/HEAD/luo_rudy_1994.cellml
		colegrove_albrecht_friel_2000/rawfile/HEAD/colegrove_albrecht_friel_2000.cellml
		dougherty_wright_yew_2005/rawfile/HEAD/dougherty_wright_yew_2005.cellml
		yamaguchi_takaki_matsubara_yasuhara_suga_1996/rawfile/HEAD/yamaguchi_takaki_matsubara_yasuhara_suga_1996.cellml
		boyett_zhang_garny_holden_2001/rawfile/HEAD/boyett_zhang_garny_holden_2001.cellml
		iribe_kohl_noble_2006/rawfile/HEAD/iribe_kohl_noble_2006.cellml
		izakov_katsnelson_blyakhman_markhasin_shkylar_1991/rawfile/HEAD/izakov_katsnelson_blyakhman_markhasin_shkylar_1991.cellml
		stern_song_sham_yang_boheler_rios_1999/rawfile/HEAD/stern_song_sham_yang_boheler_rios_1999.cellml
		devries_sherman_2000/rawfile/HEAD/devries_sherman_2000.cellml
		marhl_haberichter_brumen_heinrich_2000/rawfile/HEAD/marhl_haberichter_brumen_heinrich_2000.cellml
81	luo-rudy model	difrancesco_noble_1985/rawfile/HEAD/difrancesco_noble_1985.cellml
82	cancer metabolism	baylor_hollingworth_chandler_2002/rawfile/HEAD/baylor_hollingworth_chandler_2002_b.cellml
		baylor_hollingworth_chandler_2002/rawfile/HEAD/baylor_hollingworth_chandler_2002_d.cellml
		luo_rudy_1994/rawfile/HEAD/luo_rudy_1994.cellml
		colegrove_albrecht_friel_2000/rawfile/HEAD/colegrove_albrecht_friel_2000.cellml
		dougherty_wright_yew_2005/rawfile/HEAD/dougherty_wright_yew_2005.cellml
		yamaguchi_takaki_matsubara_yasuhara_suga_1996/rawfile/HEAD/yamaguchi_takaki_matsubara_yasuhara_suga_1996.cellml
		boyett_zhang_garny_holden_2001/rawfile/HEAD/boyett_zhang_garny_holden_2001.cellml
		iribe_kohl_noble_2006/rawfile/HEAD/iribe_kohl_noble_2006.cellml
		izakov_katsnelson_blyakhman_markhasin_shkylar_1991/rawfile/HEAD/izakov_katsnelson_blyakhman_markhasin_shkylar_1991.cellml
		stern_song_sham_yang_boheler_rios_1999/rawfile/HEAD/stern_song_sham_yang_boheler_rios_1999.cellml
		devries_sherman_2000/rawfile/HEAD/devries_sherman_2000.cellml
		marhl_haberichter_brumen_heinrich_2000/rawfile/HEAD/marhl_haberichter_brumen_heinrich_2000.cellml
		baylor_hollingworth_chandler_2002/rawfile/HEAD/baylor_hollingworth_chandler_2002_b.cellml

83	protein regulation

		baylor_hollingworth_chandler_2002/rawfile/HEAD/baylor_hollingworth_chandler_2002_d.cellml
		luo_rudy_1994/rawfile/HEAD/luo_rudy_1994.cellml
		colegrove_albrecht_friel_2000/rawfile/HEAD/colegrove_albrecht_friel_2000.cellml
		dougherty_wright_yew_2005/rawfile/HEAD/dougherty_wright_yew_2005.cellml
		yamaguchi_takaki_matsubara_yasuhara_suga_1996/rawfile/HEAD/yamaguchi_takaki_matsubara_yasuhara_suga_1996.cellml
		boyett_zhang_garny_holden_2001/rawfile/HEAD/boyett_zhang_garny_holden_2001.cellml
		iribe_kohl_noble_2006/rawfile/HEAD/iribe_kohl_noble_2006.cellml
		izakov_katsnelson_blyakhman_markhasin_shkylar_1991/rawfile/HEAD/izakov_katsnelson_blyakhman_markhasin_shkylar_1991.cellml
		stern_song_sham_yang_boheler_rios_1999/rawfile/HEAD/stern_song_sham_yang_boheler_rios_1999.cellml
		devries_sherman_2000/rawfile/HEAD/devries_sherman_2000.cellml
		marhl_haberichter_brumen_heinrich_2000/rawfile/HEAD/marhl_haberichter_brumen_heinrich_2000.cellml
84	interaction of glycolysis and mitochondrial respiration in metabolic oscillations of pancreatic islets	570/rawfile/HEAD/bertram_satin_pedersen_luciani_sherman_2007.cellml
85	luo rudy model	baylor_hollingworth_chandler_2002/rawfile/HEAD/baylor_hollingworth_chandler_2002_b.cellml
		baylor_hollingworth_chandler_2002/rawfile/HEAD/baylor_hollingworth_chandler_2002_d.cellml
		luo_rudy_1994/rawfile/HEAD/luo_rudy_1994.cellml
		colegrove_albrecht_friel_2000/rawfile/HEAD/colegrove_albrecht_friel_2000.cellml
		dougherty_wright_yew_2005/rawfile/HEAD/dougherty_wright_yew_2005.cellml
		yamaguchi_takaki_matsubara_yasuhara_suga_1996/rawfile/HEAD/yamaguchi_takaki_matsubara_yasuhara_suga_1996.cellml
		boyett_zhang_garny_holden_2001/rawfile/HEAD/boyett_zhang_garny_holden_2001.cellml
		iribe_kohl_noble_2006/rawfile/HEAD/iribe_kohl_noble_2006.cellml
		izakov_katsnelson_blyakhman_markhasin_shkylar_1991/rawfile/HEAD/izakov_katsnelson_blyakhman_markhasin_shkylar_1991.cellml
		stern_song_sham_yang_boheler_rios_1999/rawfile/HEAD/stern_song_sham_yang_boheler_rios_1999.cellml
		devries_sherman_2000/rawfile/HEAD/devries_sherman_2000.cellml
		marhl_haberichter_brumen_heinrich_2000/rawfile/HEAD/marhl_haberichter_brumen_heinrich_2000.cellml
86	di francesco-noble purkinje fibre model 1985	difrancesco_noble_1985/rawfile/HEAD/difrancesco_noble_1985.cellml
87	computational models of ventricular	iyer_mazhari_winslow_2004/rawfile/HEAD/iyer_mazhari_winslow_2004.cellml
88	cardiac	baylor_hollingworth_chandler_2002/rawfile/HEAD/baylor_hollingworth_chandler_2002_b.cellml
		baylor_hollingworth_chandler_2002/rawfile/HEAD/baylor_hollingworth_chandler_2002_d.cellml
		luo_rudy_1994/rawfile/HEAD/luo_rudy_1994.cellml
		colegrove_albrecht_friel_2000/rawfile/HEAD/colegrove_albrecht_friel_2000.cellml
		dougherty_wright_yew_2005/rawfile/HEAD/dougherty_wright_yew_2005.cellml
		yamaguchi_takaki_matsubara_yasuhara_suga_1996/rawfile/HEAD/yamaguchi_takaki_matsubara_yasuhara_suga_1996.cellml
		boyett_zhang_garny_holden_2001/rawfile/HEAD/boyett_zhang_garny_holden_2001.cellml
		iribe_kohl_noble_2006/rawfile/HEAD/iribe_kohl_noble_2006.cellml
		izakov_katsnelson_blyakhman_markhasin_shkylar_1991/rawfile/HEAD/izakov_katsnelson_blyakhman_markhasin_shkylar_1991.cellml
		stern_song_sham_yang_boheler_rios_1999/rawfile/HEAD/stern_song_sham_yang_boheler_rios_1999.cellml
		devries_sherman_2000/rawfile/HEAD/devries_sherman_2000.cellml
		marhl_haberichter_brumen_heinrich_2000/rawfile/HEAD/marhl_haberichter_brumen_heinrich_2000.cellml
89	human ventricular	tentusscher_noble_noble_panfilov_2004/rawfile/HEAD/tentusscher_noble_noble_panfilov_2004_c.cellml
		tentusscher_noble_noble_panfilov_2004/rawfile/HEAD/tentusscher_noble_noble_panfilov_2004_b.cellml
		tentusscher_noble_noble_panfilov_2004/rawfile/HEAD/tentusscher_noble_noble_panfilov_2004_a.cellml
90	bueno-orovio-cherry-fenton model	difrancesco_noble_1985/rawfile/HEAD/difrancesco_noble_1985.cellml
91	temperature	baylor_hollingworth_chandler_2002/rawfile/HEAD/baylor_hollingworth_chandler_2002_b.cellml
		baylor_hollingworth_chandler_2002/rawfile/HEAD/baylor_hollingworth_chandler_2002_d.cellml
		luo_rudy_1994/rawfile/HEAD/luo_rudy_1994.cellml
		colegrove_albrecht_friel_2000/rawfile/HEAD/colegrove_albrecht_friel_2000.cellml
		dougherty_wright_yew_2005/rawfile/HEAD/dougherty_wright_yew_2005.cellml
		yamaguchi_takaki_matsubara_yasuhara_suga_1996/rawfile/HEAD/yamaguchi_takaki_matsubara_yasuhara_suga_1996.cellml
		boyett_zhang_garny_holden_2001/rawfile/HEAD/boyett_zhang_garny_holden_2001.cellml
		iribe_kohl_noble_2006/rawfile/HEAD/iribe_kohl_noble_2006.cellml
		izakov_katsnelson_blyakhman_markhasin_shkylar_1991/rawfile/HEAD/izakov_katsnelson_blyakhman_markhasin_shkylar_1991.cellml
		stern_song_sham_yang_boheler_rios_1999/rawfile/HEAD/stern_song_sham_yang_boheler_rios_1999.cellml
		devries_sherman_2000/rawfile/HEAD/devries_sherman_2000.cellml
		marhl_haberichter_brumen_heinrich_2000/rawfile/HEAD/marhl_haberichter_brumen_heinrich_2000.cellml
92	adenosine brain	546/rawfile/HEAD/cloutier_2009.cellml
93	sglt1	267/rawfile/HEAD/SEDML/mackenzie_1996/mackenzie_1996.cellml
		267/rawfile/HEAD/mackenzie_1996.cellml
		267/rawfile/HEAD/SEDML/eskandari_2005/eskandari_2005.cellml

		267/rawfile/HEAD/eskandari_2005.cellml
		267/rawfile/HEAD/mackenzie_1996-mouse-baso.cellml
94	a model of cardiac electrical activity incorporating ionic pumps and concentration changes	difrancesco_noble_1985/rawfile/HEAD/difrancesco_noble_1985.cellml
95	oxidative phosphorylation	baylor_hollingworth_chandler_2002/rawfile/HEAD/baylor_hollingworth_chandler_2002_b.cellml
		baylor_hollingworth_chandler_2002/rawfile/HEAD/baylor_hollingworth_chandler_2002_d.cellml
		luo_rudy_1994/rawfile/HEAD/luo_rudy_1994.cellml
		colegrove_albrecht_friel_2000/rawfile/HEAD/colegrove_albrecht_friel_2000.cellml
		dougherty_wright_yew_2005/rawfile/HEAD/dougherty_wright_yew_2005.cellml
		yamaguchi_takaki_matsubara_yasuhara_suga_1996/rawfile/HEAD/yamaguchi_takaki_matsubara_yasuhara_suga_1996.cellml
		boyett_zhang_garny_holden_2001/rawfile/HEAD/boyett_zhang_garny_holden_2001.cellml
		iribe_kohl_noble_2006/rawfile/HEAD/iribe_kohl_noble_2006.cellml
		546/rawfile/HEAD/cloutier_2009.cellml
		izakov_katsnelson_blyakhman_markhasin_shkylar_1991/rawfile/HEAD/izakov_katsnelson_blyakhman_markhasin_shkylar_1991.cellml
		stern_song_sham_yang_boheler_rios_1999/rawfile/HEAD/stern_song_sham_yang_boheler_rios_1999.cellml
		devries_sherman_2000/rawfile/HEAD/devries_sherman_2000.cellml
		marhl_haberichter_brumen_heinrich_2000/rawfile/HEAD/marhl_haberichter_brumen_heinrich_2000.cellml
96	rat	baylor_hollingworth_chandler_2002/rawfile/HEAD/baylor_hollingworth_chandler_2002_b.cellml
		baylor_hollingworth_chandler_2002/rawfile/HEAD/baylor_hollingworth_chandler_2002_d.cellml
		luo_rudy_1994/rawfile/HEAD/luo_rudy_1994.cellml
		colegrove_albrecht_friel_2000/rawfile/HEAD/colegrove_albrecht_friel_2000.cellml
		dougherty_wright_yew_2005/rawfile/HEAD/dougherty_wright_yew_2005.cellml
		yamaguchi_takaki_matsubara_yasuhara_suga_1996/rawfile/HEAD/yamaguchi_takaki_matsubara_yasuhara_suga_1996.cellml
		boyett_zhang_garny_holden_2001/rawfile/HEAD/boyett_zhang_garny_holden_2001.cellml
		iribe_kohl_noble_2006/rawfile/HEAD/iribe_kohl_noble_2006.cellml
		izakov_katsnelson_blyakhman_markhasin_shkylar_1991/rawfile/HEAD/izakov_katsnelson_blyakhman_markhasin_shkylar_1991.cellml
		stern_song_sham_yang_boheler_rios_1999/rawfile/HEAD/stern_song_sham_yang_boheler_rios_1999.cellml
		devries_sherman_2000/rawfile/HEAD/devries_sherman_2000.cellml
		marhl_haberichter_brumen_heinrich_2000/rawfile/HEAD/marhl_haberichter_brumen_heinrich_2000.cellml
97	glucose	baylor_hollingworth_chandler_2002/rawfile/HEAD/baylor_hollingworth_chandler_2002_b.cellml
		baylor_hollingworth_chandler_2002/rawfile/HEAD/baylor_hollingworth_chandler_2002_d.cellml
		267/rawfile/HEAD/eskandari_2005.cellml
		luo_rudy_1994/rawfile/HEAD/luo_rudy_1994.cellml
		colegrove_albrecht_friel_2000/rawfile/HEAD/colegrove_albrecht_friel_2000.cellml
		dougherty_wright_yew_2005/rawfile/HEAD/dougherty_wright_yew_2005.cellml
		yamaguchi_takaki_matsubara_yasuhara_suga_1996/rawfile/HEAD/yamaguchi_takaki_matsubara_yasuhara_suga_1996.cellml
		boyett_zhang_garny_holden_2001/rawfile/HEAD/boyett_zhang_garny_holden_2001.cellml
		267/rawfile/HEAD/SEDML/eskandari_2005/eskandari_2005.cellml
		570/rawfile/HEAD/bertram_satin_pedersen_luciani_sherman_2007.cellml
		iribe_kohl_noble_2006/rawfile/HEAD/iribe_kohl_noble_2006.cellml
		izakov_katsnelson_blyakhman_markhasin_shkylar_1991/rawfile/HEAD/izakov_katsnelson_blyakhman_markhasin_shkylar_1991.cellml
		stern_song_sham_yang_boheler_rios_1999/rawfile/HEAD/stern_song_sham_yang_boheler_rios_1999.cellml
		devries_sherman_2000/rawfile/HEAD/devries_sherman_2000.cellml
		marhl_haberichter_brumen_heinrich_2000/rawfile/HEAD/marhl_haberichter_brumen_heinrich_2000.cellml
98	pancreatic alpha cells	baylor_hollingworth_chandler_2002/rawfile/HEAD/baylor_hollingworth_chandler_2002_b.cellml
		baylor_hollingworth_chandler_2002/rawfile/HEAD/baylor_hollingworth_chandler_2002_d.cellml
		luo_rudy_1994/rawfile/HEAD/luo_rudy_1994.cellml
		colegrove_albrecht_friel_2000/rawfile/HEAD/colegrove_albrecht_friel_2000.cellml
		dougherty_wright_yew_2005/rawfile/HEAD/dougherty_wright_yew_2005.cellml
		yamaguchi_takaki_matsubara_yasuhara_suga_1996/rawfile/HEAD/yamaguchi_takaki_matsubara_yasuhara_suga_1996.cellml
		boyett_zhang_garny_holden_2001/rawfile/HEAD/boyett_zhang_garny_holden_2001.cellml
		iribe_kohl_noble_2006/rawfile/HEAD/iribe_kohl_noble_2006.cellml
		izakov_katsnelson_blyakhman_markhasin_shkylar_1991/rawfile/HEAD/izakov_katsnelson_blyakhman_markhasin_shkylar_1991.cellml
		stern_song_sham_yang_boheler_rios_1999/rawfile/HEAD/stern_song_sham_yang_boheler_rios_1999.cellml
		devries_sherman_2000/rawfile/HEAD/devries_sherman_2000.cellml
		marhl_haberichter_brumen_heinrich_2000/rawfile/HEAD/marhl_haberichter_brumen_heinrich_2000.cellml
		iyer_mazhari_winslow_2004/rawfile/HEAD/iyer_mazhari_winslow_2004.cellml
		baylor_hollingworth_chandler_2002/rawfile/HEAD/baylor_hollingworth_chandler_2002_b.cellml

		tentusscher_noble_noble_panfilov_2004/rawfile/HEAD/tentusscher_noble_noble_panfilov_2004_b.cellml
		baylor_hollingworth_chandler_2002/rawfile/HEAD/baylor_hollingworth_chandler_2002_d.cellml
		faber_rudy_2000/rawfile/HEAD/faber_rudy_2000.cellml
		luo_rudy_1994/rawfile/HEAD/luo_rudy_1994.cellml
		colegrove_albrecht_friel_2000/rawfile/HEAD/colegrove_albrecht_friel_2000.cellml
		55c/rawfile/HEAD/Hinch_et_al_2004.cellml
		dougherty_wright_yew_2005/rawfile/HEAD/dougherty_wright_yew_2005.cellml
		556/rawfile/HEAD/niederer_hunter_smith_2006.cellml
		yamaguchi_takaki_matsubara_yasuhara_suga_1996/rawfile/HEAD/yamaguchi_takaki_matsubara_yasuhara_suga_1996.cellml
		boyett_zhang_garny_holden_2001/rawfile/HEAD/boyett_zhang_garny_holden_2001.cellml
		tentusscher_noble_noble_panfilov_2004/rawfile/HEAD/tentusscher_noble_noble_panfilov_2004_a.cellml
		tentusscher_noble_noble_panfilov_2004/rawfile/HEAD/tentusscher_noble_noble_panfilov_2004_c.cellml
		terkildsen_niederer_crampin_hunter_smith_2008/rawfile/HEAD/Hinch_et_al_2004.cellml
		hinch_greenstein_tanskanen_xu_winslow_2004/rawfile/HEAD/hinch_greenstein_tanskanen_xu_winslow_2004.cellml
		iribe_kohl_noble_2006/rawfile/HEAD/iribe_kohl_noble_2006.cellml
		shannon_wang_puglisi_weber_bers_2004/rawfile/HEAD/shannon_wang_puglisi_weber_bers_2004_a.cellml
		niederer_hunter_smith_2006/rawfile/HEAD/niederer_hunter_smith_2006.cellml
		izakov_katsnelson_blyakhman_markhasin_shkylar_1991/rawfile/HEAD/izakov_katsnelson_blyakhman_markhasin_shkylar_1991.cellml
		563/rawfile/HEAD/saucerman_brunton_michailova_mcculloch_2003.cellml
		stern_song_sham_yang_boheler_rios_1999/rawfile/HEAD/stern_song_sham_yang_boheler_rios_1999.cellml
		shannon_wang_puglisi_weber_bers_2004/rawfile/HEAD/shannon_wang_puglisi_weber_bers_2004_b.cellml
		devries_sherman_2000/rawfile/HEAD/devries_sherman_2000.cellml
		marhl_haberichter_brumen_heinrich_2000/rawfile/HEAD/marhl_haberichter_brumen_heinrich_2000.cellml
100	network	baylor_hollingworth_chandler_2002/rawfile/HEAD/baylor_hollingworth_chandler_2002_b.cellml
		baylor_hollingworth_chandler_2002/rawfile/HEAD/baylor_hollingworth_chandler_2002_d.cellml
		luo_rudy_1994/rawfile/HEAD/luo_rudy_1994.cellml
		colegrove_albrecht_friel_2000/rawfile/HEAD/colegrove_albrecht_friel_2000.cellml
		dougherty_wright_yew_2005/rawfile/HEAD/dougherty_wright_yew_2005.cellml
		yamaguchi_takaki_matsubara_yasuhara_suga_1996/rawfile/HEAD/yamaguchi_takaki_matsubara_yasuhara_suga_1996.cellml
		boyett_zhang_garny_holden_2001/rawfile/HEAD/boyett_zhang_garny_holden_2001.cellml
		iribe_kohl_noble_2006/rawfile/HEAD/iribe_kohl_noble_2006.cellml
		izakov_katsnelson_blyakhman_markhasin_shkylar_1991/rawfile/HEAD/izakov_katsnelson_blyakhman_markhasin_shkylar_1991.cellml
		stern_song_sham_yang_boheler_rios_1999/rawfile/HEAD/stern_song_sham_yang_boheler_rios_1999.cellml
		devries_sherman_2000/rawfile/HEAD/devries_sherman_2000.cellml
		marhl_haberichter_brumen_heinrich_2000/rawfile/HEAD/marhl_haberichter_brumen_heinrich_2000.cellml
101	graph	baylor_hollingworth_chandler_2002/rawfile/HEAD/baylor_hollingworth_chandler_2002_b.cellml
		baylor_hollingworth_chandler_2002/rawfile/HEAD/baylor_hollingworth_chandler_2002_d.cellml
		luo_rudy_1994/rawfile/HEAD/luo_rudy_1994.cellml
		colegrove_albrecht_friel_2000/rawfile/HEAD/colegrove_albrecht_friel_2000.cellml
		dougherty_wright_yew_2005/rawfile/HEAD/dougherty_wright_yew_2005.cellml
		yamaguchi_takaki_matsubara_yasuhara_suga_1996/rawfile/HEAD/yamaguchi_takaki_matsubara_yasuhara_suga_1996.cellml
		boyett_zhang_garny_holden_2001/rawfile/HEAD/boyett_zhang_garny_holden_2001.cellml
		iribe_kohl_noble_2006/rawfile/HEAD/iribe_kohl_noble_2006.cellml
		izakov_katsnelson_blyakhman_markhasin_shkylar_1991/rawfile/HEAD/izakov_katsnelson_blyakhman_markhasin_shkylar_1991.cellml
		stern_song_sham_yang_boheler_rios_1999/rawfile/HEAD/stern_song_sham_yang_boheler_rios_1999.cellml
		devries_sherman_2000/rawfile/HEAD/devries_sherman_2000.cellml
		marhl_haberichter_brumen_heinrich_2000/rawfile/HEAD/marhl_haberichter_brumen_heinrich_2000.cellml
102	mouse ventricular myocyte action potential calcium transients cpvt	563/rawfile/HEAD/saucerman_brunton_michailova_mcculloch_2003.cellml
103	growth hormone	baylor_hollingworth_chandler_2002/rawfile/HEAD/baylor_hollingworth_chandler_2002_b.cellml
		baylor_hollingworth_chandler_2002/rawfile/HEAD/baylor_hollingworth_chandler_2002_d.cellml
		luo_rudy_1994/rawfile/HEAD/luo_rudy_1994.cellml
		colegrove_albrecht_friel_2000/rawfile/HEAD/colegrove_albrecht_friel_2000.cellml
		dougherty_wright_yew_2005/rawfile/HEAD/dougherty_wright_yew_2005.cellml
		yamaguchi_takaki_matsubara_yasuhara_suga_1996/rawfile/HEAD/yamaguchi_takaki_matsubara_yasuhara_suga_1996.cellml
		boyett_zhang_garny_holden_2001/rawfile/HEAD/boyett_zhang_garny_holden_2001.cellml
		iribe_kohl_noble_2006/rawfile/HEAD/iribe_kohl_noble_2006.cellml
		izakov_katsnelson_blyakhman_markhasin_shkylar_1991/rawfile/HEAD/izakov_katsnelson_blyakhman_markhasin_shkylar_1991.cellml
		stern_song_sham_yang_boheler_rios_1999/rawfile/HEAD/stern_song_sham_yang_boheler_rios_1999.cellml
		devries_sherman_2000/rawfile/HEAD/devries_sherman_2000.cellml

		marhl_haberichter_brumen_heinrich_2000/rawfile/HEAD/marhl_haberichter_brumen_heinrich_2000.cellml
104	shorten wall	baylor_hollingworth_chandler_2002/rawfile/HEAD/baylor_hollingworth_chandler_2002_b.cellml
		baylor_hollingworth_chandler_2002/rawfile/HEAD/baylor_hollingworth_chandler_2002_d.cellml
		luo_rudy_1994/rawfile/HEAD/luo_rudy_1994.cellml
		colegrove_albrecht_friel_2000/rawfile/HEAD/colegrove_albrecht_friel_2000.cellml
		dougherty_wright_yew_2005/rawfile/HEAD/dougherty_wright_yew_2005.cellml
		yamaguchi_takaki_matsubara_yasuhara_suga_1996/rawfile/HEAD/yamaguchi_takaki_matsubara_yasuhara_suga_1996.cellml
		boyett_zhang_garny_holden_2001/rawfile/HEAD/boyett_zhang_garny_holden_2001.cellml
		iribe_kohl_noble_2006/rawfile/HEAD/iribe_kohl_noble_2006.cellml
		shorten_robson_mckinnon_wall_2000/rawfile/HEAD/shorten_robson_mckinnon_wall_2000.cellml
		izakov_katsnelson_blyakhman_markhasin_shkylar_1991/rawfile/HEAD/izakov_katsnelson_blyakhman_markhasin_shkylar_1991.cellml
		stern_song_sham_yang_boheler_rios_1999/rawfile/HEAD/stern_song_sham_yang_boheler_rios_1999.cellml
		devries_sherman_2000/rawfile/HEAD/devries_sherman_2000.cellml
		marhl_haberichter_brumen_heinrich_2000/rawfile/HEAD/marhl_haberichter_brumen_heinrich_2000.cellml
105	zhang h, holden av, kodama i, honjo h, lei m, varghese t, boyett mr	baylor_hollingworth_chandler_2002/rawfile/HEAD/baylor_hollingworth_chandler_2002_b.cellml
		baylor_hollingworth_chandler_2002/rawfile/HEAD/baylor_hollingworth_chandler_2002_d.cellml
		luo_rudy_1994/rawfile/HEAD/luo_rudy_1994.cellml
		colegrove_albrecht_friel_2000/rawfile/HEAD/colegrove_albrecht_friel_2000.cellml
		dougherty_wright_yew_2005/rawfile/HEAD/dougherty_wright_yew_2005.cellml
		yamaguchi_takaki_matsubara_yasuhara_suga_1996/rawfile/HEAD/yamaguchi_takaki_matsubara_yasuhara_suga_1996.cellml
		boyett_zhang_garny_holden_2001/rawfile/HEAD/boyett_zhang_garny_holden_2001.cellml
		iribe_kohl_noble_2006/rawfile/HEAD/iribe_kohl_noble_2006.cellml
		izakov_katsnelson_blyakhman_markhasin_shkylar_1991/rawfile/HEAD/izakov_katsnelson_blyakhman_markhasin_shkylar_1991.cellml
		stern_song_sham_yang_boheler_rios_1999/rawfile/HEAD/stern_song_sham_yang_boheler_rios_1999.cellml
		devries_sherman_2000/rawfile/HEAD/devries_sherman_2000.cellml
		marhl_haberichter_brumen_heinrich_2000/rawfile/HEAD/marhl_haberichter_brumen_heinrich_2000.cellml
106	a synthetic oscillatory network of transciptional regulators	baylor_hollingworth_chandler_2002/rawfile/HEAD/baylor_hollingworth_chandler_2002_b.cellml
		baylor_hollingworth_chandler_2002/rawfile/HEAD/baylor_hollingworth_chandler_2002_d.cellml
		luo_rudy_1994/rawfile/HEAD/luo_rudy_1994.cellml
		colegrove_albrecht_friel_2000/rawfile/HEAD/colegrove_albrecht_friel_2000.cellml
		dougherty_wright_yew_2005/rawfile/HEAD/dougherty_wright_yew_2005.cellml
		yamaguchi_takaki_matsubara_yasuhara_suga_1996/rawfile/HEAD/yamaguchi_takaki_matsubara_yasuhara_suga_1996.cellml
		boyett_zhang_garny_holden_2001/rawfile/HEAD/boyett_zhang_garny_holden_2001.cellml
		iribe_kohl_noble_2006/rawfile/HEAD/iribe_kohl_noble_2006.cellml
		izakov_katsnelson_blyakhman_markhasin_shkylar_1991/rawfile/HEAD/izakov_katsnelson_blyakhman_markhasin_shkylar_1991.cellml
		stern_song_sham_yang_boheler_rios_1999/rawfile/HEAD/stern_song_sham_yang_boheler_rios_1999.cellml
		devries_sherman_2000/rawfile/HEAD/devries_sherman_2000.cellml
		marhl_haberichter_brumen_heinrich_2000/rawfile/HEAD/marhl_haberichter_brumen_heinrich_2000.cellml
107	a model of the ventricular cardiac action	luo_rudy_1991/rawfile/HEAD/luo_rudy_1991.cellml
108	the ord human ventricular action potential model	w/andre/SAN-ORd/rawfile/HEAD/Ohara_Rudy_2011.cellml
109	computational model for emergent dynamics in the heart	baylor_hollingworth_chandler_2002/rawfile/HEAD/baylor_hollingworth_chandler_2002_b.cellml
		baylor_hollingworth_chandler_2002/rawfile/HEAD/baylor_hollingworth_chandler_2002_d.cellml
		luo_rudy_1994/rawfile/HEAD/luo_rudy_1994.cellml
		colegrove_albrecht_friel_2000/rawfile/HEAD/colegrove_albrecht_friel_2000.cellml
		dougherty_wright_yew_2005/rawfile/HEAD/dougherty_wright_yew_2005.cellml
		yamaguchi_takaki_matsubara_yasuhara_suga_1996/rawfile/HEAD/yamaguchi_takaki_matsubara_yasuhara_suga_1996.cellml
		boyett_zhang_garny_holden_2001/rawfile/HEAD/boyett_zhang_garny_holden_2001.cellml
		iribe_kohl_noble_2006/rawfile/HEAD/iribe_kohl_noble_2006.cellml
		difrancesco_noble_1985/rawfile/HEAD/difrancesco_noble_1985.cellml
		izakov_katsnelson_blyakhman_markhasin_shkylar_1991/rawfile/HEAD/izakov_katsnelson_blyakhman_markhasin_shkylar_1991.cellml
		stern_song_sham_yang_boheler_rios_1999/rawfile/HEAD/stern_song_sham_yang_boheler_rios_1999.cellml
		devries_sherman_2000/rawfile/HEAD/devries_sherman_2000.cellml
		marhl_haberichter_brumen_heinrich_2000/rawfile/HEAD/marhl_haberichter_brumen_heinrich_2000.cellml
110	a model of excitation and adaptation in bacterial chemotaxis	baylor_hollingworth_chandler_2002/rawfile/HEAD/baylor_hollingworth_chandler_2002_b.cellml
		baylor_hollingworth_chandler_2002/rawfile/HEAD/baylor_hollingworth_chandler_2002_d.cellml
		luo_rudy_1994/rawfile/HEAD/luo_rudy_1994.cellml
		colegrove_albrecht_friel_2000/rawfile/HEAD/colegrove_albrecht_friel_2000.cellml
		dougherty_wright_yew_2005/rawfile/HEAD/dougherty_wright_yew_2005.cellml
		yamaguchi_takaki_matsubara_yasuhara_suga_1996/rawfile/HEAD/yamaguchi_takaki_matsubara_yasuhara_suga_1996.cellml

		boyett_zhang_garny_holden_2001/rawfile/HEAD/boyett_zhang_garny_holden_2001.cellml
		iribe_kohl_noble_2006/rawfile/HEAD/iribe_kohl_noble_2006.cellml
		izakov_katsnelson_blyakhman_markhasin_shkylar_1991/rawfile/HEAD/izakov_katsnelson_blyakhman_markhasin_shkylar_1991.cellml
		stern_song_sham_yang_boheler_rios_1999/rawfile/HEAD/stern_song_sham_yang_boheler_rios_1999.cellml
		devries_sherman_2000/rawfile/HEAD/devries_sherman_2000.cellml
		marhl_haberichter_brumen_heinrich_2000/rawfile/HEAD/marhl_haberichter_brumen_heinrich_2000.cellml

Table S3. The best multipliers setup and results. We examine three feature modifications, i.e. without modification (WPURE), adding the preferred label to other features (WPL), and adding the preferred label to empty features only (WPLE). We also examine three additional scenarios to calculate the degree of association, i.e. considering all features with dependency level (mode_1), considering all features without dependency level (mode_2), and considering one feature only from ontology dictionaries with the highest weight combined with description feature from the model entity and dependency level (mode_3).

Feature modification	Similarity measure	Parser	(α β γ θ δ)	AUC_{PR}
wple	mode_1	benepar	(3.0, 0.0, 0.0, 0.0, 0.81)	0.387
		stanza	(3.0, 0.4, 0.0, 0.0, 0.13)	0.452
		coreNLP	(3.0, 0.0, 0.0, 0.0, 0.16)	0.528
		xStanza	(3.0, 0.4, 0.0, 0.0, 0.26)	0.472
	mode_2	benepar	(3.0, 0.0, 0.0, 0.0, 1.41)	0.344
		stanza	(3.0, 0.7, 0.0, 0.0, 2.67)	0.333
		coreNLP	(3.0, 1.0, 0.3, 0.3, 1.67)	0.405
		xStanza	(3.0, 0.0, 0.0, 0.0, 0.19)	0.371
	mode_3	benepar	(3.0, 0.5, 0.0, 0.1, 0.86)	0.39
		stanza	(3.0, 0.5, 0.0, 0.0, 0.21)	0.454
		coreNLP	(3.0, 0.5, 0.0, 0.0, 0.31)	0.517
		xStanza	(3.0, 0.5, 0.0, 0.0, 0.21)	0.474
wpl	mode_1	benepar	(3.0, 1.1, 0.0, 0.4, 1.12)	0.395
		stanza	(3.0, 0.4, 0.0, 0.2, 0.12)	**0.463**
		coreNLP	(3.0, 0.4, 0.0, 0.2, 0.21)	0.546
		xStanza	(3.0, 0.3, 0.1, 0.2, 0.12)	0.476
	mode_2	benepar	(3.0, 0.9, 0.0, 0.0, 1.8)	0.362
		stanza	(3.0, 0.7, 0.0, 0.0, 2.67)	0.332
		coreNLP	(3.0, 1.0, 0.0, 0.3, 2.0)	0.421
		xStanza	(3.0, 0.1, 0.7, 0.1, 0.29)	0.376
	mode_3	benepar	(3.0, 3.0, 0.0, 0.0, 1.5)	**0.408**
		stanza	(3.0, 0.5, 0.0, 0.1, 0.11)	**0.463**
		coreNLP	(3.0, 3.0, 0.0, 0.0, 0.38)	**0.553**
		xStanza	(3.0, 1.0, 0.1, 0.2, 0.12)	**0.478**
wpure	mode_1	benepar	(3.0, 0.0, 0.0, 0.0, 0.81)	0.387
		stanza	(3.0, 0.0, 0.0, 0.0, 0.16)	0.441
		coreNLP	(3.0, 0.0, 0.0, 0.0, 0.16)	0.528
		xStanza	(3.0, 0.0, 0.0, 0.0, 0.16)	0.457
	mode_2	benepar	(3.0, 0.0, 0.0, 0.0, 1.41)	0.344
		stanza	(3.0, 0.6, 0.0, 0.3, 2.4)	0.33
		coreNLP	(3.0, 0.0, 0.2, 0.0, 1.5)	0.402
		xStanza	(3.0, 0.0, 0.0, 0.0, 0.19)	0.371
	mode_3	benepar	(3.0, 0.9, 0.0, 0.0, 0.94)	0.372
		stanza	(3.0, 0.5, 0.0, 0.0, 0.21)	0.409
		coreNLP	(3.0, 0.5, 0.0, 0.0, 0.43)	0.477
		xStanza	(3.0, 0.6, 0.2, 0.0, 0.6)	0.436

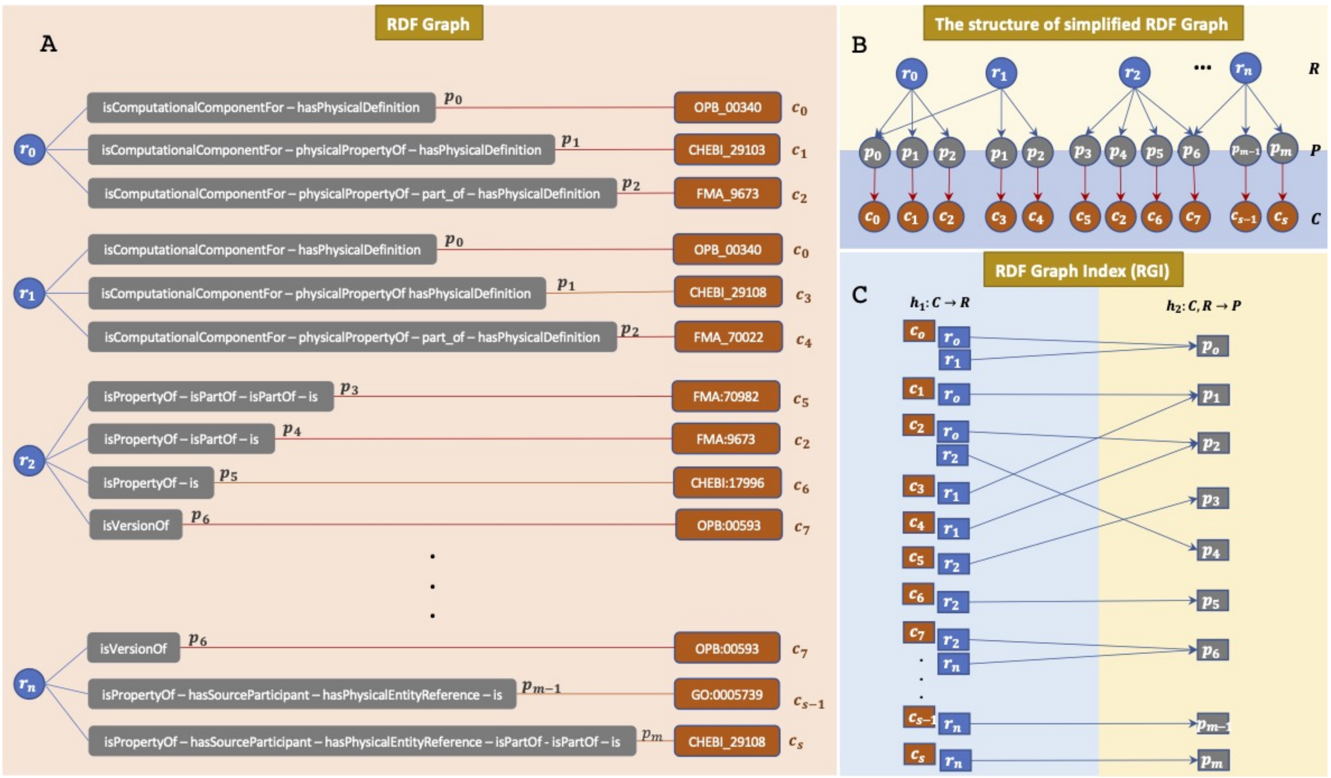

Figure S1: The creation of the RDF Graph Index (RGI). (A) The structure of RDF annotation in repositories. There are multiple trees with entities as roots R, ontology classes C describing entities R, and paths P defining how ontology classes C describing entities R. (B) Since a pair of path and ontology class can appear in multiple trees, the structure of the RDF Graph can be simplified by providing a single entry for each pair referenced by multiple roots $\bar{R}$. (C) RDF Graph Index (RGI) consisting of indexes mapping ontology classes C to entities R and (C, R) to paths P.

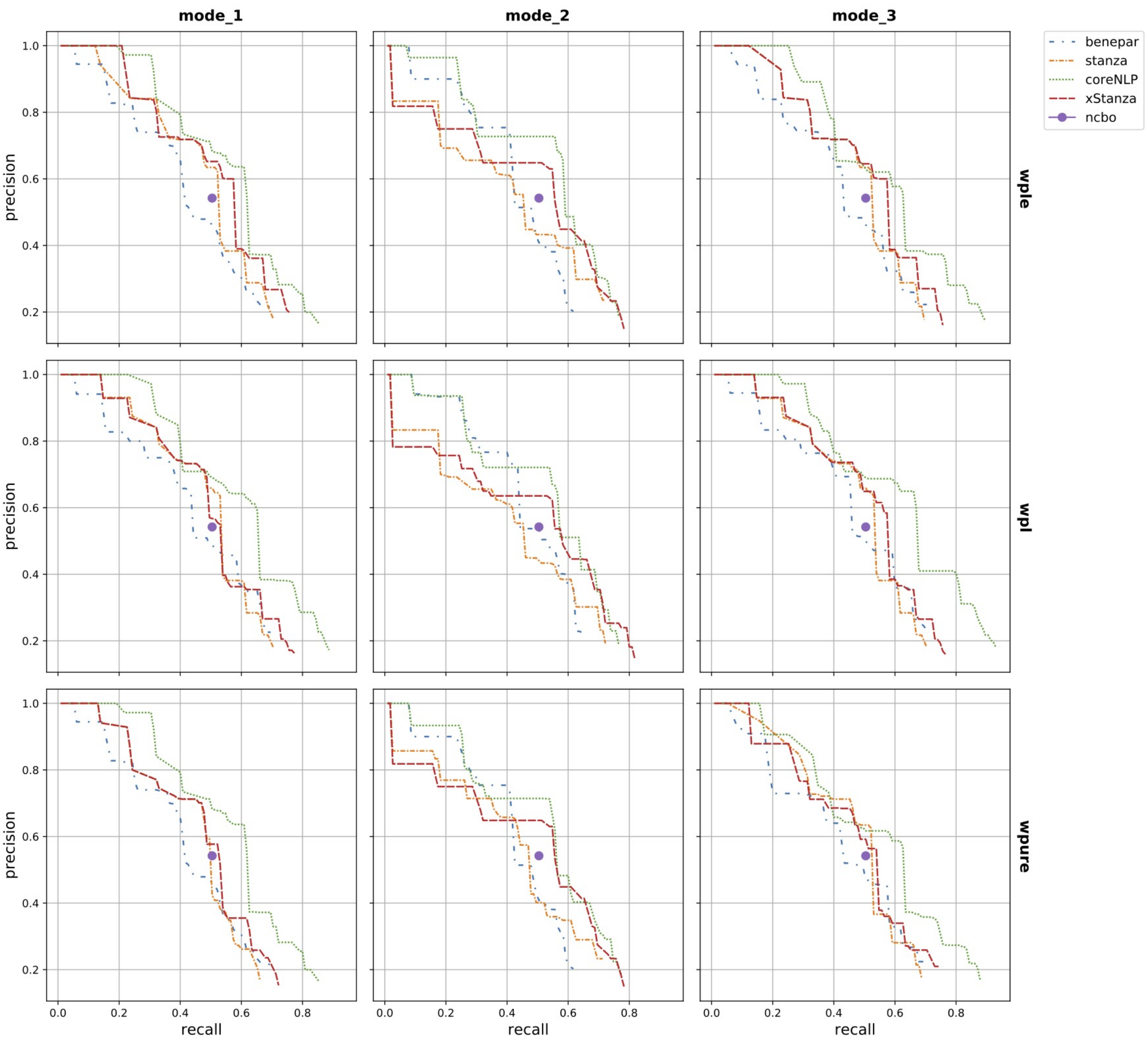

Figure S2: The performance of NLIMED to annotate NLQ on a test data containing 52 NLQ. The use of CoreNLP demonstrates the best AuC_{PR} (Precision-Recall AuC, a single value representing the combination of different precision and recall pairs), followed by xStanza. Compared to NCBO Annotator where its precision and recall is 0.542 and 0.504, respectively, almost all parsers perform better. Interpolated AuC_{PR} of NLIMED differentiate with four parsers (CoreNLP, Benepar, Stanza, xStanza), three feature modifications (WPURE, WPL, WPLE), and three scenarios to calculate the degree of association (mode_1, mode_2, mode_3).

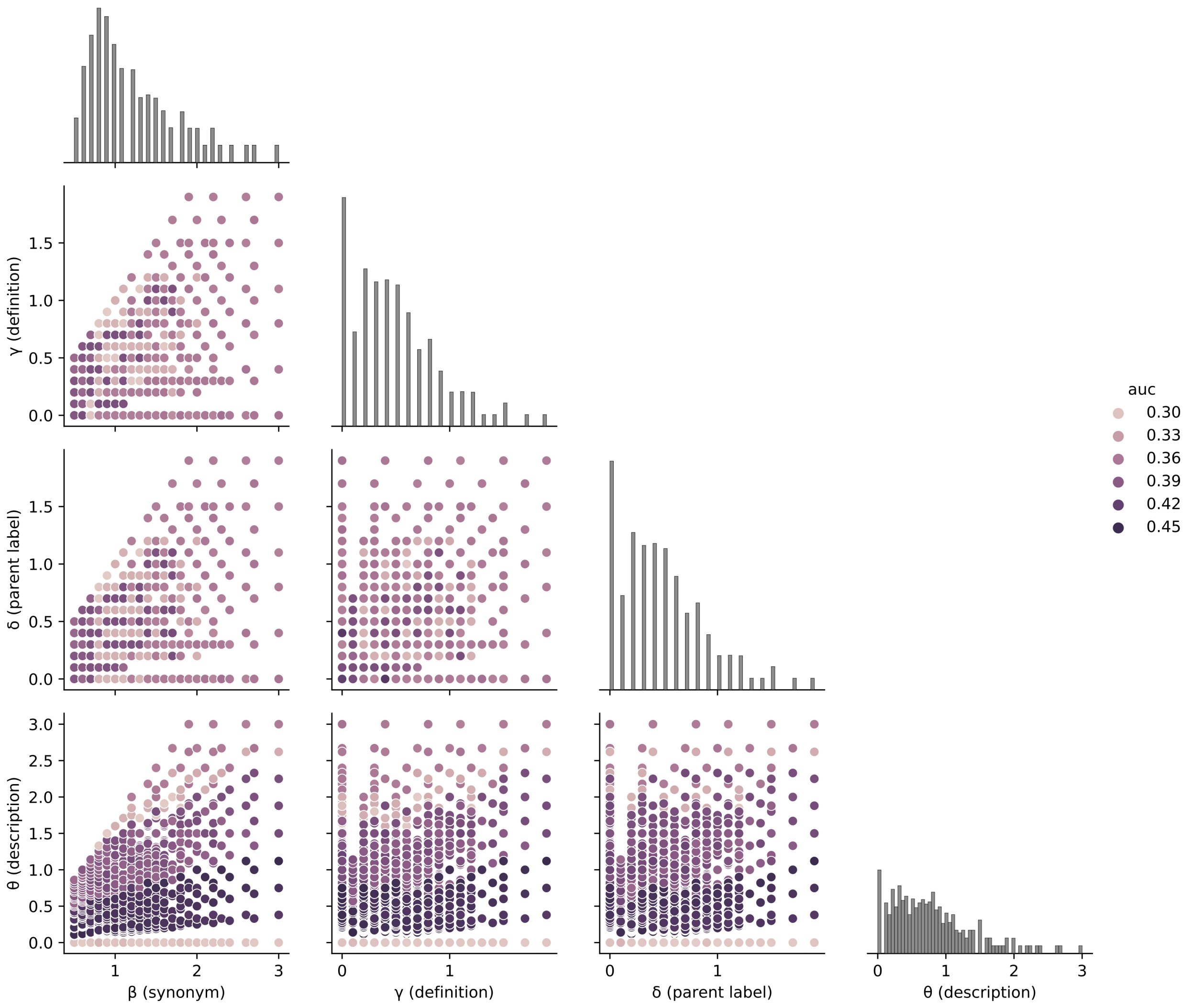

Figure S3: The interaction of features and their role in annotating a query to ontology classes using Stanza, WPL, mode_3, and α=3.0. The most important feature is the preferred label followed by the description and synonym, whereas the parent label and definition have the least contributions.

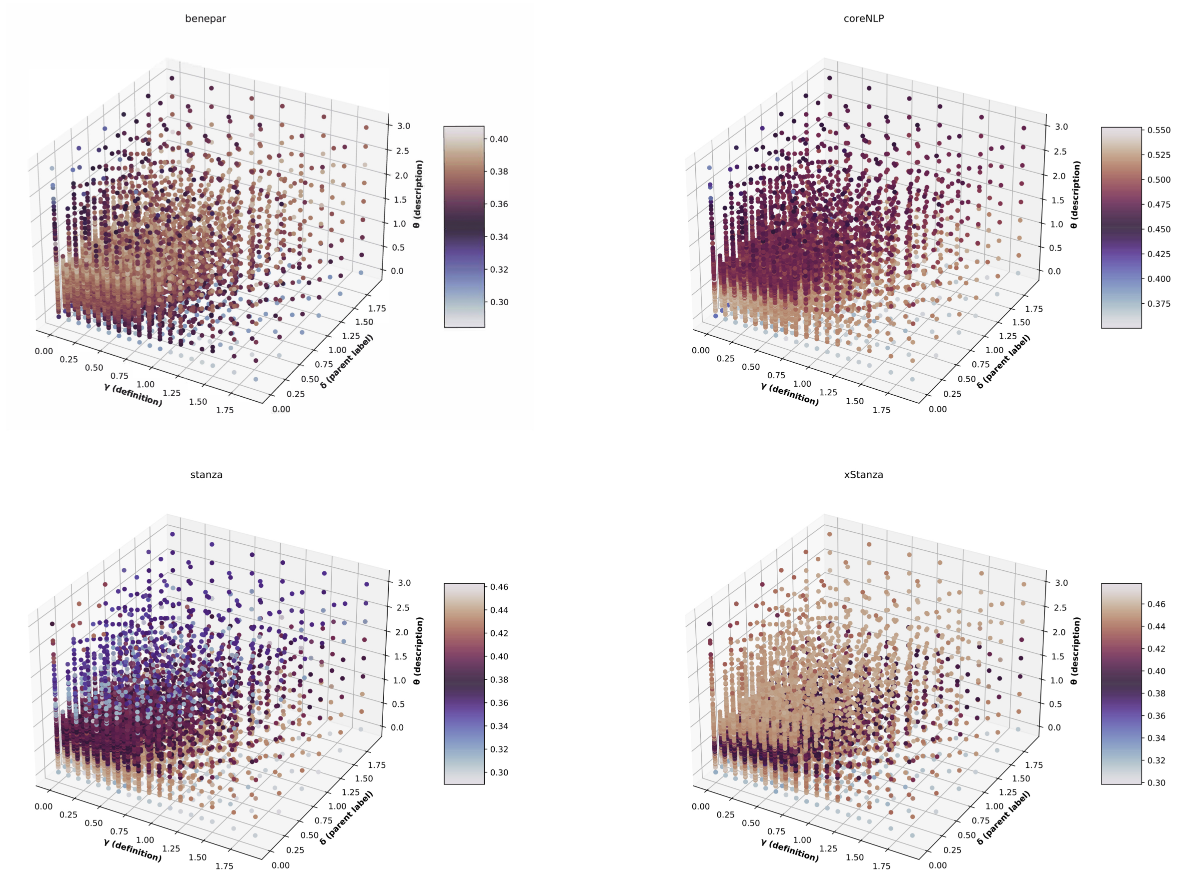

Figure S4: The interaction of γ(definition), δ(parent label), and θ(description) using wpl and mode_3 along with their AuC_{PR}

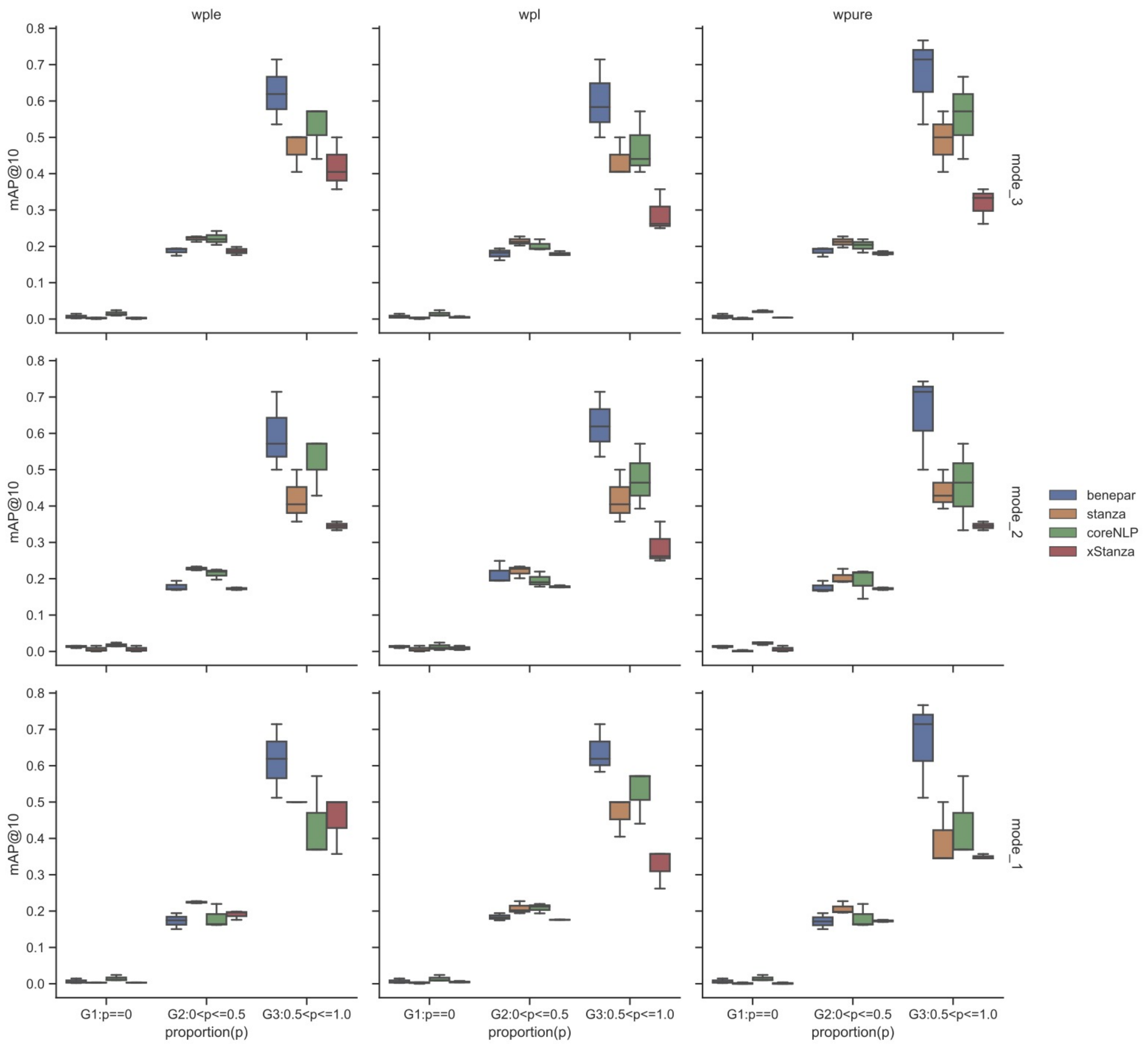

Figure S5: The mAP@10 of NLIMED on historical query-results records in the PMR differentiate with the highest proportion of terms in one of their ontology classes appear in the query. The multiplier combinations used are the best combination stated at Subsection **??** with additional combinations of α, β, γ, δ, and θ as (3.0, 3.0, 0.5, 0.5, 0.5) and (3.0, 3.0, 1.0, 1.0, 1.0).
